# Genome-wide analysis of local chromatin packing in *Arabidopsis thaliana*

**DOI:** 10.1101/gr.170332.113

**Published:** 2015-02

**Authors:** Congmao Wang, Chang Liu, Damian Roqueiro, Dominik Grimm, Rebecca Schwab, Claude Becker, Christa Lanz, Detlef Weigel

**Affiliations:** 1 Department of Molecular Biology, Max Planck Institute for Developmental Biology, 72076 Tübingen, Germany;; 2 Machine Learning and Computational Biology Research Group, Max Planck Institute for Developmental Biology and Max Planck Institute for Intelligent Systems, 72076 Tübingen, Germany

## Abstract

The spatial arrangement of interphase chromosomes in the nucleus is important for gene expression and genome function in animals and in plants. The recently developed Hi-C technology is an efficacious method to investigate genome packing. Here we present a detailed Hi-C map of the three-dimensional genome organization of the plant *Arabidopsis thaliana*. We find that local chromatin packing differs from the patterns seen in animals, with kilobasepair-sized segments that have much higher intrachromosome interaction rates than neighboring regions, representing a dominant local structural feature of genome conformation in *A. thaliana*. These regions, which appear as positive strips on two-dimensional representations of chromatin interaction, are enriched in epigenetic marks H3K27me3, H3.1, and H3.3. We also identify more than 400 insulator-like regions. Furthermore, although topologically associating domains (TADs), which are prominent in animals, are not an obvious feature of *A. thaliana* genome packing, we found more than 1000 regions that have properties of TAD boundaries, and a similar number of regions analogous to the interior of TADs. The insulator-like, TAD-boundary-like, and TAD-interior-like regions are each enriched for distinct epigenetic marks and are each correlated with different gene expression levels. We conclude that epigenetic modifications, gene density, and transcriptional activity combine to shape the local packing of the *A. thaliana* nuclear genome.

The spatial organization of the genome in the nucleus is critical for many cellular processes (Van Bortle and Corces 2012). It has been broadly accepted that the packing of chromatin inside the nucleus is not random, but structured at several hierarchical levels (Gibcus and Dekker 2013). Cytological studies in different species have revealed that each chromosome typically occupies a distinct domain within the nucleus, and the arrangement of these chromosome territories with respect to each other appears to be largely stable during the interphase of the cell cycle.

Using chromatin conformation capture (3C) methods, it has been possible to break the resolution barrier of microscopy-based approaches and to map spatial interactions between specific genomic loci at sub-kilobasepair scales (Dekker et al. 2002). More recently, combination of 3C principles with massive parallel sequencing has enabled the simultaneous interrogation of many interacting loci throughout the genome (de Wit and de Laat 2012). Among these, the Hi-C approach combines affinity purification and next-generation sequencing to capture genome-wide DNA linkages (Lieberman-Aiden et al. 2009). The increase in resolution afforded by these methods has substantially advanced our knowledge of chromatin arrangement in three-dimensional (3D) space and, for example, shown that topologically associating domains (TADs) are a structural feature of several metazoan genomes (Dekker et al. 2013). Each TAD is a relatively isolated local packing unit, such that long-range interactions between loci within one TAD are strongly preferred over those between different TADs. The median sizes of TADs are around 900 kb in mice and humans and 60 kb in fruit flies (Dixon et al. 2012; Hou et al. 2012). Despite the difference in size, TADs are reflected in local gene expression, and, at least in animals, by the epigenetic landscape and the binding of insulator proteins (Lam et al. 2009; Dixon et al. 2012; Hou et al. 2012; Sexton et al. 2012). At the megabase-scale, TAD patterns are largely conserved between different cell lines and even across species (Dixon et al. 2012). At smaller scales, TADs can split or merge in response to developmental cues (Phillips-Cremins et al. 2013).

What we know about the packing of chromosomes in plant nuclei comes mostly from cytological studies, using either in situ hybridization of fixed material or fluorescently marked transgene insertions in live cells (Schubert and Shaw 2011; Tiang et al. 2012). These experiments have indicated that the overall interphase chromosome conformation differs between species. Species with chromosomes longer than 500 Mb tend to adopt a “Rabl” configuration (Rabl 1885), in which centromeres and telomeres are located at opposite poles of the nuclei (Fransz and de Jong 2011). Smaller chromosomes, such as those of *A. thaliana*, which are only in the range of 20 Mb, adopt a rosette configuration, with chromocenters that contain the densely packed centromeres and other large repeats and with euchromatic loops that emanate from the chromocenters (Fransz et al. 2002). The size of the euchromatic loops for the same region of the genome can vary, but it has been proposed that their formation reflects interstitial blocks of heterochromatic regions that are incorporated into the chromocenters (Fransz et al. 2002). The relative positioning of chromosomal territories in *A. thaliana* nuclei appears to be largely random, with the exception that the nucleolus organizing region (NOR)-bearing Chromosomes 2 and 4 associate more often with each other than expected by chance (Pecinka et al. 2004). The subnuclear positioning of *A. thaliana* genes inside nuclei can be linked to gene expression, as deduced from a survey of tagged transgene insertions (Rosin et al. 2008), and Polycomb-silenced alleles may also be found in similar locations (Rosa et al. 2013).

For plants, the Hi-C technique was first employed to demonstrate global decondensation of heterochromatin as a consequence of *MICRORCHIDIA6* (*MORC6*) inactivation (Moissiard et al. 2012), while the 4C method has been used to determine genome-wide interactions of 13 individual loci (Grob et al. 2013). Recent Hi-C experiments with a six-cutter enzyme further revealed strong long-range tethering among several heterochromatic islands throughout the *A. thaliana* genome (Feng et al. 2014; Grob et al. 2014). Here, we use Hi-C to study the local packing of the *A. thaliana* genome at higher resolution with a four-cutter enzyme. In contrast to animals, we did not find evidence for a prevalent partitioning of *A. thaliana* chromatin into TADs. Instead, we identified many local regions that we call positive strips, which stand out because they interact much more frequently than the genome-wide average with other sequences. We also identified hundreds of insulator-like regions and regions analogous to TAD boundaries or TAD internal sequences. These regions are associated with various genomic features such as epigenetic modifications and gene and transcription activity. Together, our work provides a framework in which the structural changes during evolution of plant genomes can be studied.

## Results

### Hi-C map resolution

The generation of a Hi-C map begins with digestion of chromatin with restriction enzymes. Very different from mammalian genomes with transcription units being dozens or hundreds of kilobasepairs (kb) long, the *A. thaliana* genome has a much higher gene density, with an average of 4.4 kb per gene. If TAD size was governed by the number of genes in each TAD, *A. thaliana* would be expected to have much smaller TADs than mammals, necessitating increased Hi-C map resolution. Since the Hi-C detection of DNA interactions relies on cutting at least once between interacting sites, the resolution of a Hi-C map is determined by a combination of average restriction fragment size and sequencing depth. We therefore compared Hi-C results after digestion with a four-cutter enzyme, DpnII, and a six-cutter enzyme, HindIII. Complete digestion of the *A. thaliana* reference genome with DpnII should generate more than 432,000 fragments with a median size of 169 bp. However, as cross-linked chromatin will interfere with enzymatic digestion, many potential cut sites should be blocked. Indeed, we found that digested DNA fragments were shifted toward larger molecular sizes, with the bulk ranging from 200 to 1000 bp (Supplemental Fig. 1). In negative control experiments, we performed cross-link reversal before ligation, which leads to self-ligation products of incompletely digested DNA fragments and ligation products from random interactions between distant DNA fragments (Supplemental Fig. 2A). The negative control Hi-C map featured strong local signals from the self-ligation products and a largely uniform genome-wide background from the random interactions (Supplemental Fig. 3). As expected, the DpnII four-cutter enzyme greatly reduced the length of incomplete digestion products (Supplemental Fig. 2B), with only ∼3% of control reads indicative of DNA loops between 2 and 50 kb. For the HindIII six-cutter, the rate was ∼45% (Supplemental Fig. 2C). Thus, four-cutter enzymes should support the analysis of DNA interactions with kb resolution.

### Correlation of Hi-C interactions with known genome features

We analyzed two biological replicates with DpnII and a single sample with HindIII, all collected from 10-d-old seedlings. After filtering and elimination of duplicate reads, the data sets contained between 24 and 68 million reads (Supplemental Table 1). We normalized each data set by taking potential technical biases into account (Yaffe and Tanay 2011) and produced Hi-C maps at various resolutions (see details in Methods). The reads from DpnII-treated samples allowed us to identify statistically significant contacts up to a distance of 60 kb at 2-kb resolution (Supplemental Fig. 4). All three Hi-C data sets were strongly correlated with each other (Supplemental Fig. 5; Supplemental Table 2). The overall patterns were similar to a recently published Hi-C map produced with HindIII, which was based on 21 million informative reads (Supplemental Fig. 5; Moissiard et al. 2012).

**Figure 1.**
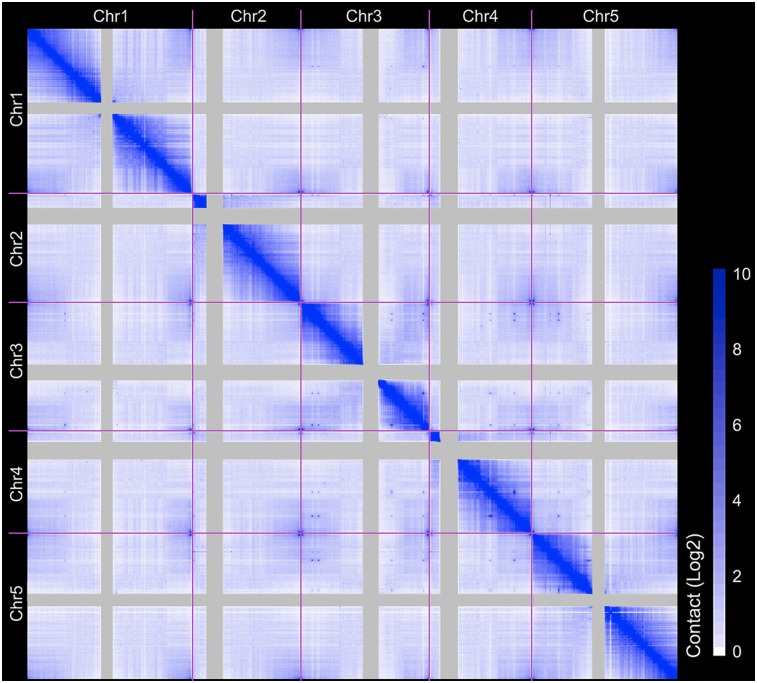
Genome-wide interaction map of *A. thaliana* at 20-kb resolution. Elements represent normalized contact strength. Centromeric regions are masked.

Because *A. thaliana* is a selfer and the material analyzed was homozygous throughout the genome, our Hi-C analyses could not separate interactions within the same chromosome molecule from those between homologous chromosomes. We refer to both types of interaction as intrachromosomal interactions. Most interactions were within chromosomes, consistent with the known chromosome territories; in addition, interactions within chromosome arms were preferred (Fig. 1; Supplemental Fig. 6). In agreement with previous fluorescent in situ hybridization (FISH) studies (Fransz et al. 2002), all telomeres interacted with each other, except for those from the short arms of Chromosomes 2 and 4 (Fig. 1; Supplemental Fig. 7). Instead, the telomeres close to the NORs on the short arms of Chromosomes 2 and 4 were frequently associated with centromeric regions (Supplemental Fig. 8). Our data sets also support many strong long-range intra- and interchromosomal interactions that have been reported elsewhere (Fig. 1; Feng et al. 2014; Grob et al. 2014).

### Hi-C interactions and epigenetic marks

**Figure 2.**
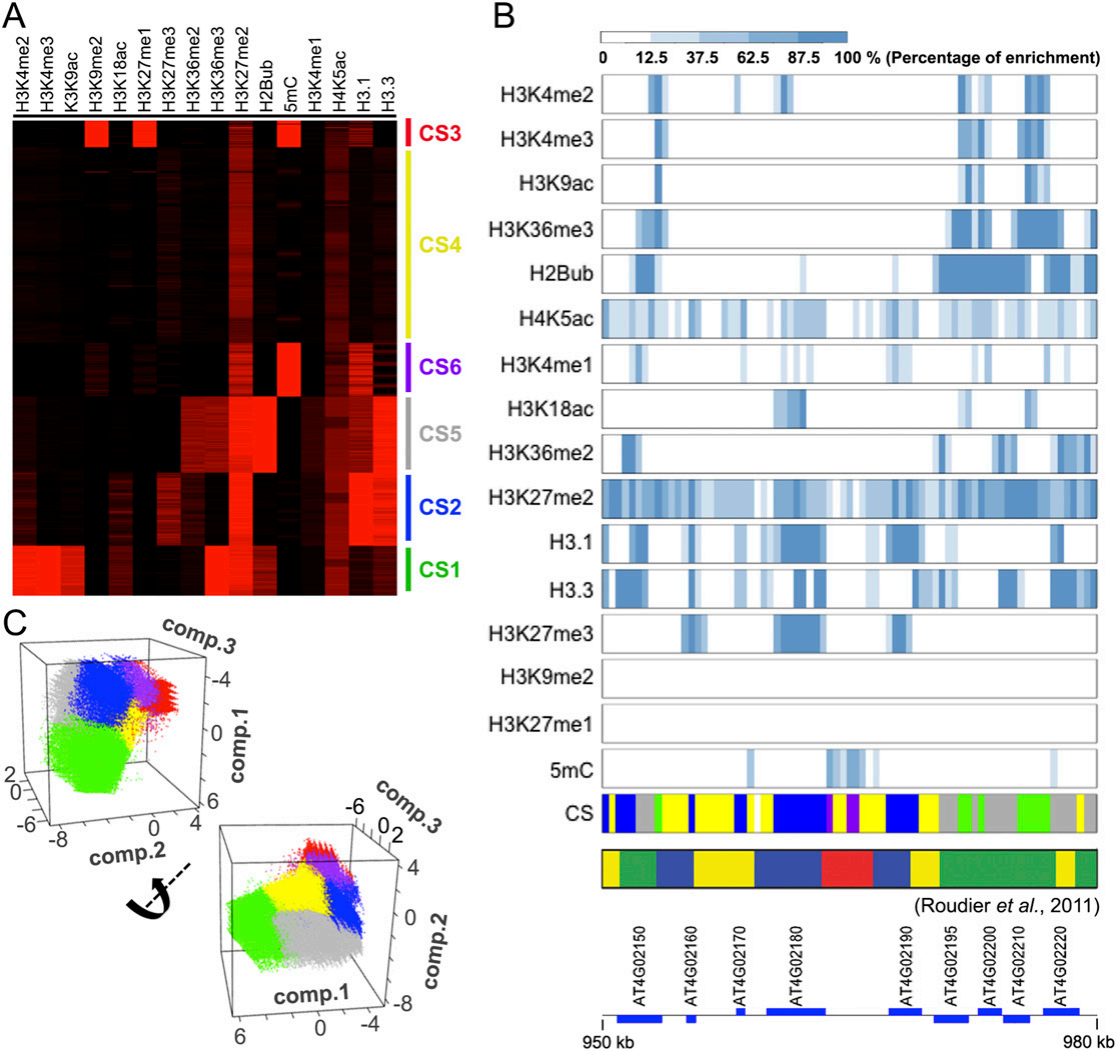
Classification of the *A. thaliana* epigenome at 400-bp resolution. (*A*) Identification of chromatin states (CS). CS1–4 are according to Roudier et al. (2011), who used 12 epigenetic data sets that partially overlap with the 16 data sets used here. CS5 (gray) is related to CS1 (green) and CS6 (purple) to CS3 (red). (*B*) Enrichment of 16 epigenetic marks and chromatin state classification in 400-bp bins in a region from Chromosome 4. Comparison of CS classification in this study and that by Roudier et al. (2011) is also shown. (*C*) Visualization of classified bins with the first three principal components. Bins are colored according to their CS groups.

In animals, local chromatin packing reflects specific epigenetic marks (Lieberman-Aiden et al. 2009; Dixon et al. 2012; Hou et al. 2012; Sexton et al. 2012). For this purpose, we asked whether epigenetic marks also correlated with features of our Hi-C maps. In *A. thaliana*, chromatin occurs in four main states characterized by different ensembles of epigenetic marks (Roudier et al. 2011). We used published data for 13 histone modifications and two histone variants (Zhang et al. 2009; Costas et al. 2011; Roudier et al. 2011; Luo et al. 2012; Park et al. 2012; Stroud et al. 2012), along with a new set of DNA methylation data, all of which were collected from tissues similar to those used in our Hi-C experiments (Fig. 2A; Supplemental Tables 3, 4; see details in Methods). These 16 epigenetic marks partially overlapped with the 12 marks used by Roudier et al. (2011) to classify Chromosome 4; with 400-bp-size bins, our genome-wide clustering gave similar results (Fig. 2A,B). We further subdivided the transcriptionally active euchromatin into chromatin state 1 (CS1) and CS5, and the classical heterochromatin into CS3 and CS6 (Fig. 2A). CS5 was even more enriched for H3K36me2, H2Bub, and H3.3 than CS1, but had less H3K4me2, H3K4me3, or H3K9ac. CS6 was distinguished from CS3 by having less H3K9me2 or H3K27me1. CS2 was enriched for H3K27me3, H3.1, and H3.3. CS4 showed no particular enrichment for any marks (Fig. 2A). In a principal component analysis (PCA), euchromatic CS1 and CS5 were well separated from heterochromatic CS3 and CS6, with CS2 and CS4 being intermediate (Fig. 2C). Larger bins of 2 kb, but not 5 kb, produced similar results (Supplemental Fig. 9; Supplemental Table 5).

**Figure 3.**
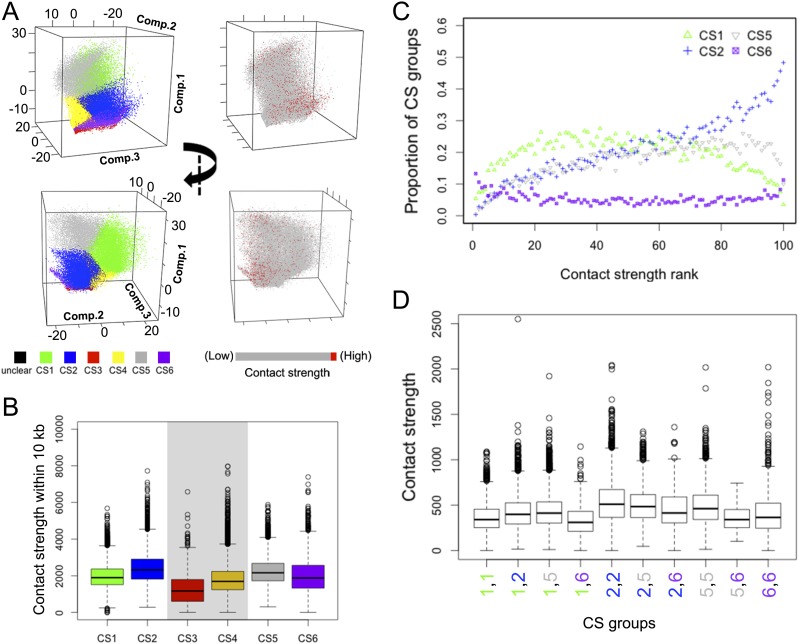
Epigenetic marks and contact strength. (*A*) Comparison of CS and contact strength across 2-kb bins graphed according to the first three principal components for epigenetic marks (similar to Fig. 2C). Contact strength is the sum of contacts of a 2-kb bin with the 10 surrounding bins. “Unclear” indicates no enrichment for any epigenetic mark. (*B*) Contact strengths of bins according to CS classification. CS3 and CS4 are shaded, as their sequences display biases that might result in lower contact strength values. (*C*) CS classification of bins ranked by contact strength, from low to high. (*D*) Contact strength of adjacent bins. In *C,D*, due to biases, data regarding CS3 and CS4 are not shown, but they are presented in Supplemental Figure 12. In *B–D*, the few bins of unclear categorization are not shown.

To capture local interactions, we calculated “contact strength” as the sum of contacts of each 2-kb bin with neighboring bins (within 10 kb), and assessed how CS affected this measure. Because CS3 and CS4 bins had sequence biases, which might result in lower sequencing coverage and contact strength (Supplemental Fig. 10), they were not included in the comparison. We found that bins in the top 5% most often had CS2 (Fig. 3A; Supplemental Fig. 11). Generally, CS2 had higher contact strength than others (Fig. 3B,C; Supplemental Fig. 12A). The association between contact strength and individual epigenetic marks generally reflected the marks typical for the different CS classes (Supplemental Fig. 13). Similar patterns were found when correlating epigenetic profiles with contact strengths at distances of 10 to 20 kb and above 20 kb (data not shown).

Approaching associations from the other side, we found that local interactions of CS2 with other bins were the strongest, with the highest mean value for CS2/CS2 interactions (Fig. 3D; Supplemental Fig. 12B). For bins 100 kb apart, interactions between two CS6 bins were the strongest (Supplemental Fig. 12C). Together, these results suggest that chromatin contacts over short and long distances are either guided by epigenetic marks, or conversely, that chromatin contacts affect epigenetic marks.

### Local chromatin packing features

**Figure 4.**
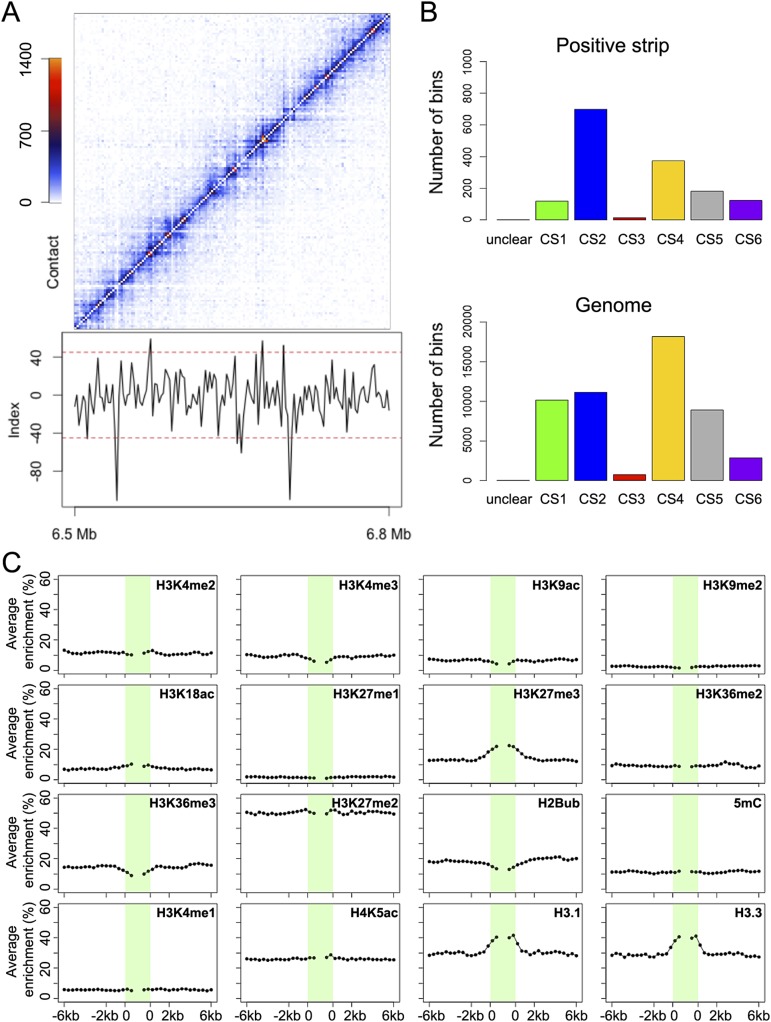
Genomic features associated with local strips. (*A*) Hi-C map of a genomic region from Chromosome 1. Index used to quantify local contrast is plotted *below*. Red dashed lines depict thresholds for calling strips. (*B*) Annotation of bins forming positive strips by CS. (*C*) Epigenetic marks around positive strips. Average enrichment means the percentage of each 400-bp region claimed as enriched for the respective epigenetic mark.

Next, we asked whether TADs, as in mammals and *Drosophila*, could be detected in *A. thaliana* Hi-C data. At resolutions of 2 or 20 kb, neither the DpnII nor the HindIII Hi-C maps showed TADs as prevailing features (Supplemental Fig. 14; Fig. 4A; data not shown). Instead, bins that had either excessive or strongly depressed contacts with surrounding bins stood out at 2-kb resolution (Fig. 4A; Supplemental Fig. 14C), with similar patterns in two biological replicates (Supplemental Fig. 15). The two types of bins, with excessive or strongly depressed contacts, sharply differed from surrounding bins and thus formed contrasting lines that we named positive and negative strips (Supplemental Table 6; see details in Methods). Because sequences in bins forming negative strips suffered from sequencing bias, we excluded them from further analysis (Supplemental Fig. 16).

Across a host of genomic features, positive strips were only marginally enriched in gene bodies (Supplemental Fig. 17). Nearly half of positive strips were CS2 (698 out of 1506 2-kb bins in positive strips, *P* << 10^−10^, Fisher’s exact test) (Fig. 4B). The distribution of individual epigenetic marks around positive strips was as expected from the integrated chromatin states, with marks characteristic for CS2 being enriched around positive strips (Fig. 4C). We next asked whether positive strips and nearby gene expression affected each other. We performed RNA-seq and divided genes into nine groups according to expression levels (Supplemental Fig. 18). Genes with transcription start sites (TSSs) or gene bodies inside positive strips tended to be expressed at lower levels than average, which correlated with the enrichment of the repressive histone mark H3K27me3; on the other hand, genes transcribed away from positive strips did not show changes in distribution of expression levels (Supplemental Fig. 19).

### Insulator-like, TAD-boundary-like, and TAD-interior-like regions

**Figure 5.**
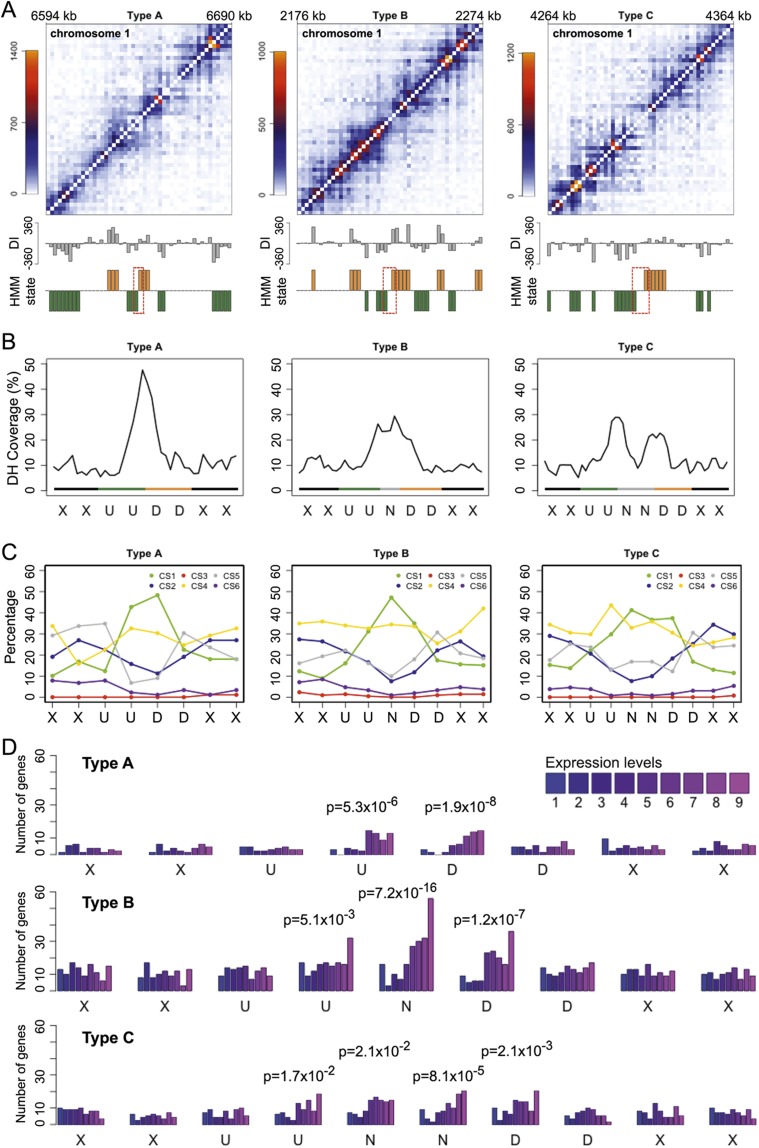
Insulator-like regions in the *A. thaliana* genome. (*A*) Examples of each type of insulator-like region, which are placed at the center of the Hi-C map details. Directionality index (DI) and HMM state are shown at the *bottom*. Positive and negative DI values indicate bins with stronger-than-expected interactions with downstream and upstream regions. Orange indicates downstream and green upstream bias, and the dashed boxes insulator-like regions. (*B*) DNase I hypersensitive sites (DH) around insulator-like regions. Coverage means the average percentage of each 500-bp bin annotated with such a feature. “U” and “D” bins have biased interactions with upstream and downstream regions, while “N” bins have no directionality bias. “X” indicates bins with any type of HMM state. (*C*) CS annotation of bins around insulator-like regions. (*D*) Distribution of genes by expression level around insulator-like regions. For bins from insulator-like regions, the *P*-values indicate the significance of change in expression level distribution from Cramér-von Mises tests. For *C* and *D*, the HMM state of bins are labeled as in *B*. See Supplemental Figure 18B for categorization of expression levels.

From the Hi-C map, we noticed regions over which interactions between the flanking regions was weaker. Because such patterns were reminiscent of insulator regions in animals, we named these regions “insulator-like.” Based on the interaction directionality bias of bins inferred from a hidden Markov model (HMM), we systematically identified more than 400 insulator-like regions in *A. thaliana* (Fig. 5A; Supplemental Fig. 20; Supplemental Table 7; see details in Methods). We found tight association between insulator-like regions and DNase I hypersensitive sites (DH) reported for leaf tissue (Zhang et al. 2012). Insulator-like regions were enriched for CS1, indicating that in general they were composed of highly accessible euchromatin (Fig. 5B,C). The distribution of individual epigenetic marks revealed enrichment of a subset of classical hallmarks of active chromatin such as H3K4me2, H3K4me3, H3K9ac, and H3K36me3 (Supplemental Figs. 21–23). As expected, genes located in insulator-like regions were highly expressed (Fig. 5D). These observations demonstrated a remarkable similarity between insulator-like regions in *A. thaliana* and TAD boundaries in animals, which are enriched for insulator binding motifs, active chromatin, and highly expressed genes (Dixon et al. 2012; Hou et al. 2012; Sexton et al. 2012).

**Figure 6.**
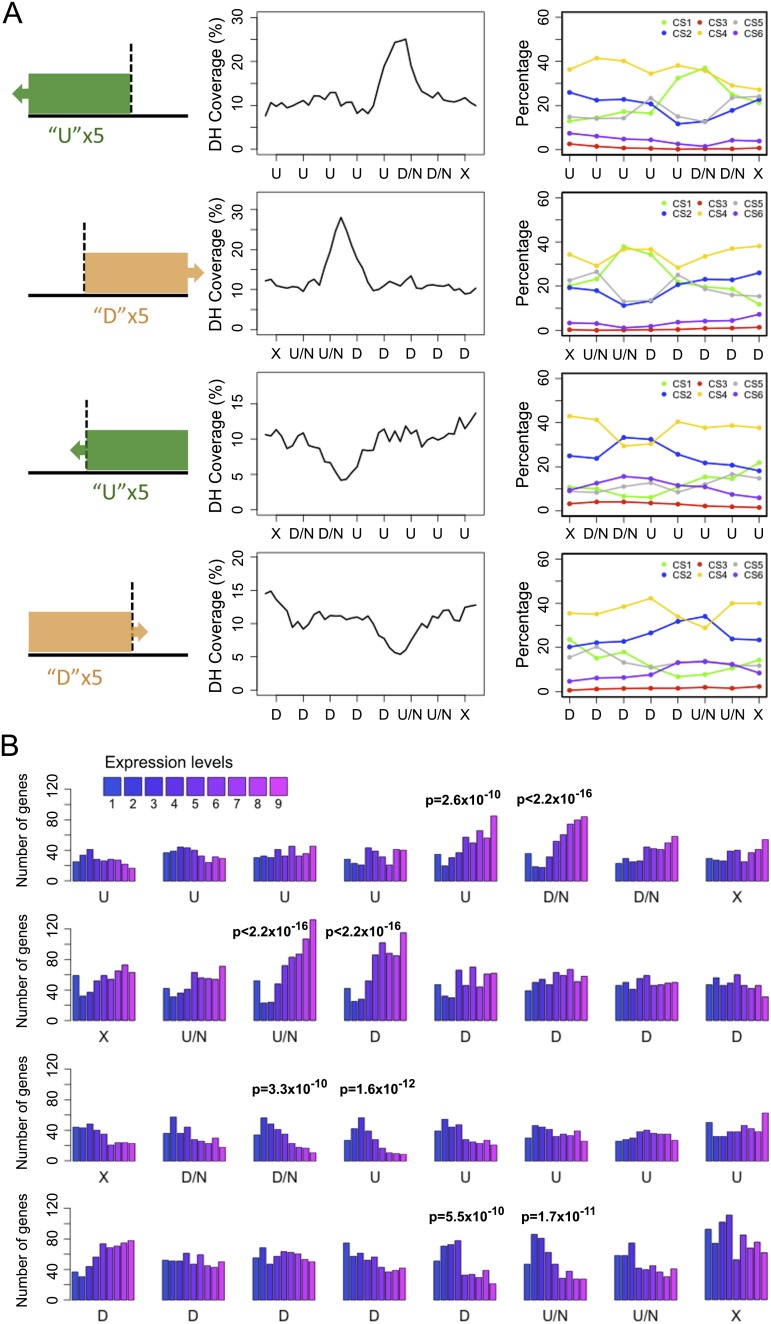
TAD-boundary-like and TAD-interior-like regions in the *A. thaliana* genome. (*A*) DNase I hypersensitive sites and CS. The cartoon on the *left* shows how a stretch of bins marked “U” or “D” is aligned with respect to the one marking either the start or the end of the pattern (highlighted with a vertical dotted line). (*B*) Genes ranked by expression level around TAD-boundary-like (*top* two rows) and TAD-interior-like regions (*bottom* two rows). Each row represents regions with bins of specific HMM state sequence. See figure legend of Figure 5B–D for other labels.

We further extended our search for TAD-boundary-like regions, which were either the starting point of a block of bins preferentially interacting with downstream regions, or the end point of a block of bins preferentially interacting with upstream regions. We termed regions marking the start of an upstream biased block or the end of a downstream biased block as “TAD-interior-like” (Fig. 6A; Supplemental Fig. 20; see details in Methods). We found more than 1000 regions for each type (Supplemental Tables 8, 9). By performing the same association analysis described for insulator-like regions, we found that TAD-boundary-like and TAD-interior-like regions had contrasting biological properties. Similar to insulator-like regions, TAD-boundary-like regions were enriched for DH sites and various activating epigenetic marks indicative of transcriptionally active euchromatin, whereas TAD-interior-like regions had opposite patterns indicative of transcriptionally repressed heterochromatin (Fig. 6; Supplemental Figs. 24–27).

## Discussion

Both short- and long-range chromatin interactions sculpt the arrangement of the genome in three-dimensional space. Our Hi-C analyses have revealed that both types of interactions correlate with specific epigenetic modifications in *A. thaliana*. At distances in the range of 100 kb and above, interaction between heterochromatic bins tended to be the strongest. These results are consistent with the focused analysis of 13 *A. thaliana* genomic regions by 4C (Grob et al. 2013). Experiments with *lacO* repeat arrays integrated in the *A. thaliana* genome have suggested that the clustering of *lacO* sequences at distant loci depended on heterochromatic marks (Jovtchev et al. 2011). The clustering of heterochromatin is in line with the observation of “AB compartments” from animal Hi-C data sets, where euchromatin and heterochromatin are spatially segregated (Lieberman-Aiden et al. 2009). Thus, long-range interactions between heterochromatic regions might be one of the main forces shaping the overall packing of chromosomes, by promoting dynamic clustering of heterochromatin, which includes the looping structures found around chromocenters (Fransz et al. 2002).

At a resolution of 2 kb, we identified specific bins that had much higher contact strengths than their neighbors, forming positive strips on a two-dimensional map. It is noteworthy that positive strips could not be detected when equal sequencing depth across the genome has to be assumed, a requirement, for example, for the ICE normalization method (Imakaev et al. 2012). Such methods were inappropriate for our data, because the size range of sonicated DNA we excluded (below 300 bp) from our Hi-C sequencing libraries greatly overlapped with that of total DNA digested by DpnII (Supplemental Fig. 1). Thus, size selection after DpnII digestion preferentially removes unligated or self-ligated DNA fragments. Therefore, chromatin with higher-than-average looping frequency produce more Hi-C reads, as they are more likely to become incorporated into a longer ligation product of chimeric DNA. As positive strips have more frequent interactions with a broad range of neighboring chromatin, we interpret them as a dynamic structural feature that can only be observed by analyzing a large number of cells. This raises the possibility that genes in these regions are more sensitive to developmental reprogramming, as they could contact more often distant regions that might contain different *cis*-regulatory elements. Positive strips were enriched for bins with a higher incidence of the (PRC2) repressor complex 2-dependent mark H3K27me3 (Schuettengruber et al. 2007; Schwartz and Pirrotta 2008). Such bins tended to have stronger interactions with nearby bins within 10 kb (Figs. 2A, 3C), suggesting that regions marked with H3K27me3 are generally “sticky” in *A. thaliana*. We also found that the largest fraction of highly significant interacting loci comprised regions enriched in H3K27me3 (CS2 bins) (Supplemental Table 10). It has been reported that *FLC-lacO*-tagged loci from two chromosome homologs tend to cluster in endoreduplicated cells as a consequence of Polycomb-mediated epigenetic silencing (Rosa et al. 2013). Thus, the “stickiness” of H3K27me3-enriched regions could be promoting clustering during Polycomb-mediated silencing. Different from what we found in *A. thaliana*, animal Hi-C maps do not indicate strong interactions among H3K27me3-enriched chromatin (Sexton et al. 2012). It is noteworthy that the genome-wide distribution of H3K27me3 in *A. thaliana* has several distinct features, including specific association with LHP1 (LIKE HETEROCHROMATIN 1), homologs of which in turn bind to methylated H3K9 in animals (Turck et al. 2007; Zhang et al. 2007). These features might be related to its biophysical properties, including higher strength of contacts with neighboring regions.

Distinct TADs are a major structural characteristic of mammalian and *Drosophila* genomes. Notably, >40% of TADs found in *Drosophila* Kc167 cells were also detected in nuclei from embryos, which represent a heterogeneous cell population (Hou et al. 2012; Sexton et al. 2012). This similarity can be partly explained by TADs being linked to static features such as gene density and distribution of insulator binding motifs (Hou et al. 2012). The pattern of mammalian genome packing is highly conserved between different cell types and across species (Dixon et al. 2012). Similar to the situation in *Drosophila*, sequence features such as insulator CTCF binding sites, housekeeping genes, tRNA genes, and SINE retrotransposons have been suggested to play roles in establishing mammalian genome packing (Dixon et al. 2012). TAD boundaries stand out as insulators, since interactions between flanking regions are suppressed. Although we did not observe TADs as a prevailing structural property of the *A. thaliana* genome, we could extract hundreds of insulator-like regions, and more than 1000 TAD-boundary-like regions, which showed local Hi-C patterns that were comparable to regions demarcating animal TADs. The presence of highly expressed genes, but not of tRNA genes and SINEs in these regions appears to be a feature shared between animals and plants (Figs. 5, 6; Supplemental Fig. 28). Although plants lack CTCF-like and other animal-typical insulator proteins (Ong and Corces 2009), AS1 (ASYMMETRIC LEAVES1) and AS2 have been proposed to function conceptually similar to genetic insulators (Guo et al. 2008). That AS1 binds only a few hundred loci in the genome (Iwasaki et al. 2013) is, however, inconsistent with a role as a general insulator. Furthermore, AS1 binding sites were not enriched in the insulator-like regions we identified in this study. If plants do use certain proteins as substitutes for animal insulator-binding proteins, the insulator-like regions identified in this work provide a potential avenue toward their identification.

TAD-interior-like regions tend to be transcriptionally less active, consistent with more tightly packed chromatin. A similar phenomenon has been noted in *Drosophila*, where inactive chromatin was mostly found within large TADs (Hou et al. 2012). It is intriguing that despite having TAD-boundary-like and TAD-interior-like regions, these are not arranged in a manner that makes for animal-like TADs in *A. thaliana*. Two possible reasons are the absence of canonical insulators that separate larger domains, or the high gene density. Our Hi-C data indicate that in addition to genomic distance, the interaction strength between two genomic fragments was associated with epigenetic modifications (Fig. 3C; Supplemental Fig. 13). We propose that above nucleosomes, regions with lengths comparable to gene bodies tend to form the next hierarchical level of structural units, and that these structural units have different activities in terms of making contacts with surrounding regions. Since most *A. thaliana* gene bodies are only a few kb in length, the mapping of such smaller units will require Hi-C maps at even higher resolution than we have provided here. Alternatively, a subset of regions could be more finely dissected and interrogated with the 5C approach, with which one can probe regions of interest with much higher sequencing depth (Dostie and Dekker 2007). In either approach, the genome would have to be fragmented into even shorter pieces than what can be achieved with four-cutter restriction enzymes.

## Methods

### Plant material

*Arabidopsis thaliana* accession Columbia (Col-0) was grown at 23°C in long days (16 h light/8 h dark) on half-strength Murashige & Skoog (MS) medium with 1% sucrose and 0.3% phytagel. The aerial portions of 10-d-old seedlings were harvested at Zeitgeber time (ZT) 6.

### Hi-C library preparation

An improved Hi-C protocol (Belton et al. 2012) was adapted for plants and for different restriction enzymes. *S*eedlings were fixed with 1% formaldehyde solution in MS buffer (10 mM potassium phosphate, pH 7.0; 50 mM NaCl; 0.1M sucrose) at room temperature for 30 min in a vacuum. After fixation, the seedlings were incubated at room temperature for 5 min under vacuum in MC buffer with 0.15 M glycine. Approximately 2 g fixed tissue was homogenized with liquid nitrogen and resuspended in nuclei isolation buffer (Louwers et al. 2009) and filtered with a 40-nm cell strainer. The procedures for enriching nuclei from flow-through and subsequent denaturation were done according to a 3C protocol established for maize (Louwers et al. 2009). The denatured chromatin was equally divided into five tubes, and each was digested with 25 units of DpnII or HindIII overnight at 37°C. On the next day, digested DNA was blunt-ended by filling nucleotides by Klenow enzyme (Belton et al. 2012), during which biotin-14-dCTP (Invitrogen) was incorporated. For DpnII, an additional incubation at 65°C for 15 min was performed before the nucleotide fill-in reaction to inactivate this restriction enzyme. Next, the chromatin solution was diluted 10 times and blunt-end ligation was carried out in three tubes at 16°C overnight, with each tube having 100 Weiss units of T4 DNA ligase. On the next day, crosslinks were reversed in one tube by 65°C-overnight incubation. The other two tubes were treated as controls; blunt-end ligation and reversal of crosslinking were performed exactly the same but with reverse order. After ligation, DNA isolation and RNA digestion were performed according to (Belton et al. 2012).

Next, from Hi-C and control samples, 3 µg of DNA were incubated with T4 DNA polymerase to remove the biotin-14-dCTP from the end of unligated DNA molecules by following procedures described in Belton et al. (2012). The protective nucleotides added were dTTP and dATP for DpnII, and dGTP and dATP for HindIII. DNA was then sheared by sonication with a Covaris S220; end repair and adenylation of DNA ends were performed as described (Lieberman-Aiden et al. 2009). Subsequently, the DNA was separated in a 1.8% agarose gel and fragments of 300–600 bp were cut out and recovered from the gel. DNA was purified with Dynabeads MyOne Streptavidin C1 beads (Invitrogen), and on-bead ligation with selected Illumina TruSeq Adaptor was done as described (Belton et al. 2012), except that 0.5 µl adaptor was used for each ligation. After washing, the beads were resuspended in 20 µl of 10 mM Tris-HCl buffer (pH 8.0).

Amplification of library molecules was performed according to the standard Illumina library preparation protocol. For a 50-µl PCR reaction, 5 µl of recovered Hi-C DNA bound to magnetic beads was used as template, and typically 10 cycles of amplification were used for Hi-C and 13 for control libraries. PCR products were purified and quantified with an Agilent 2100 Bioanalyzer, and sequenced on an Illumina HiSeq 2000 with 2× 101 bp reads.

### Read mapping and filtering

Paired reads were aligned against the *A. thaliana* genome (TAIR10) using BWA 0.6.2 with default parameters (Li and Durbin 2009). Only uniquely mapped paired reads were kept for downstream analysis. An iterative strategy (Imakaev et al. 2012) was used to improve mapping accuracy of reads containing chimeric sequences due to the presence of a ligation junction. Raw reads were trimmed to 25 bp for initial mapping; trimming of multiply mapping reads was successively reduced in 2-bp steps up to a maximum of 101 bp. After this procedure, all mapping results were merged. Subsequently, the best alignment was selected based on the highest mapping quality and longest read length.

Different criteria were applied to identify inter- and intrachromosomal interactions of reads. For each pair of reads from interchromosomal interactions, the sum of mapped read lengths, plus their distances to the first enzyme cutting site downstream should be smaller than the library insert size (600 bp). For intrachromosomal interactions, three additional filtering criteria were employed. First, only read pairs with apparent insert sizes >600 bp were kept as candidate interaction pairs if the mapped paired reads were in forward/reverse direction (i.e., Paired End mode) (Langmead et al. 2009). Second, a self-ligation filter, which checks the presence of the enzyme cutting site between mapped reads, was employed if the mapped paired reads are in reverse/forward direction (i.e., Mate Pair mode). In this case, a read pair was treated as a self-ligation product if there was no cutting site found in the genome reference sequence between mapped reads. Finally, all read pairs in Mate Pair mode with a distance between mapped loci below 1500 bp were removed, to reduce the presence of self-ligation products resulting from incompletely digested DNA fragments (Supplemental Fig. 29; Jin et al. 2013).

### Hi-C data normalization

Restriction fragment length, GC content of fragment ends, and mappability of sequence reads are the main sources of Hi-C biases (Yaffe and Tanay 2011). We further found that for each bin, the number of mappable sequences flanking restriction enzyme cutting sites imposes an additional level of bias on sequencing depth; we term this factor as fragment end count (i.e., “number of GATC ends” for Hi-C maps generated with DpnII). We used an improved HiCNorm-based method (Hu et al. 2012) to remove these biases via Poisson regression.

We assumed that 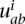 followed a Poisson distribution with rate 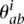:
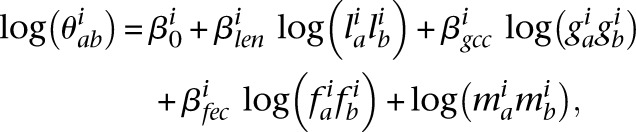
with 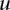 being the number of paired-end reads spanning two bins; 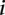 the chromosome ID; 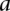 and 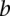, bin IDs; 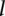, fragment length; 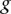, GC content feature; 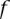, fragment end count feature; 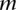, mappability feature; and 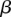, bias coefficient. The residuals of the Poisson regression were treated as the normalized Hi-C interaction matrix (Hu et al. 2012).

Fragment length and GC content were calculated as described (Yaffe and Tanay 2011), while mappability was calculated based on our iterative mapping approach. Bins were excluded from normalization if their mappability was below 0.5, or if effective fragment length was below 0.1. When filling in the normalized Hi-C matrix, zero was assigned to bins excluded in the normalization step, but they were marked and not included in downstream analyses. In addition, if not excluded due to the above-mentioned criteria, bins in the centromeric regions of each chromosome (Chr 1, 13.7–15.9 Mb; Chr 2, 2.45–5.50 Mb; Chr 3, 11.3–14.3 Mb; Chr 4, 1.80–5.15 Mb; Chr 5, 11.0–13.35 Mb) were included in normalization but excluded from the Hi-C map pattern analysis unless otherwise stated. For local Hi-C feature analysis, only reads generated from DpnII digestion were used.

### RNA-seq analysis

Total RNA was isolated with an RNeasy Plant Mini Kit (Qiagen), and libraries were prepared according to a standard protocol (Illumina). We obtained 31.5 and 54.5 million reads from the biological replicates. RNA-seq reads were aligned against the *A. thaliana* cDNA reference (TAIR10) using BWA 0.6.2 with default parameters (Li and Durbin 2009). Normalized RPKM (reads per kilobase per million mapped reads) counts for each gene were calculated (Mortazavi et al. 2008).

### Bisulfite sequencing

Two-week-old plants growing on half-strength MS plates at 23°C in long days were collected. DNA extraction, library preparation, sequencing, and read alignment were performed as described (Becker et al. 2011).

### Integration of epigenome data

Published ChIP-seq and ChIP-chip data (Zhang et al. 2009; Costas et al. 2011; Roudier et al. 2011; Luo et al. 2012; Park et al. 2012; Stroud et al. 2012) were originally mapped to the TAIR8 version of the *A. thaliana* reference genome. For ChIP-seq data, 50-bp fragments were retrieved from the TAIR8 genome and mapped to TAIR10. If a sequence fragment mapped to multiple locations on the reference genome, it was randomly assigned to one of the loci. For ChIP-chip data, each probe on the chip was mapped to TAIR10. Probes with multiple mapping locations were discarded. Where possible, we chose ChIP-seq data over ChIP-chip for the same epigenetic mark. Detailed information regarding the data sets is given in Supplemental Table 3.

Enriched regions from ChIP-seq data sets were called with SICER v1.1 (Zang et al. 2009) with FDR ≤ 0.01, while enriched probes from ChIP-chip data sets were called with the ChIPmix method as described (Roudier et al. 2011). The latter method was used to define enriched probes in the H2Bub ChIP-chip data set (Roudier et al. 2011), hence the reported enrichment results were used directly. For DNA methylation, methylated regions were defined by an HMM approach (Hagmann et al. 2015).

We partitioned the *A. thaliana* genome into 400-bp bins. For each epigenetic mark, an integer score (0 to 4) was given to every bin proportionally to the quintile of enrichment in that bin. Bins (∼3%) with assigned 0 for all 16 epigenetic marks were defined as “unclear” and were excluded from following *k*-means clustering analysis. We used the Cluster 3.0 program to cluster bins into six groups via calculating Euclidean distances, and the nomenclature of these groups was according to Roudier et al. (2011). We found that the algorithm separated classical euchromatin (CS1) and heterochromatin (CS3) defined by Roudier et al. (2011) into two subgroups, named CS5 and CS6. We applied the same strategy for 2-kb bins.

### Identification of positive and negative strips

We extracted the individual contact strength between a focal 2-kb bin and its neighboring bins at distances from 6 kb up to 50 kb (23 bins upstream and 23 bins downstream). For each contact strength value obtained from the bin of interest, a positive score was given if it was at least in the 80th percentile among the equivalent values obtained from its 200 neighbors, and this score was increased if it was at least in the 95th percentile. Negative scores were given for values up to the 20th or fifth percentile. We explored different scores and found that ±1 and ±3 gave good results that agreed well with visual inspection of the Hi-C map. All intermediate values, between the 20th and 80th percentile, were given 0, and the sum of these 46 values, which we named “index,” was used to quantitatively describe how much contrast a bin had compared to its neighbors in the context of a local 400-kb region. We set a cutoff value at ±45 to call a strip. A sketch in Supplemental Figure 30 further illustrates how the calculation was performed.

### Hidden Markov model and identification of insulator-, TAD-boundary-, and TAD-interior-like regions

We applied methods described by Dixon et al. (2012) to calculate the directionality index (DI) and HMM state of bins, excluding those not used for Hi-C map normalization or belonging to centromeric regions. For each bin, we included its interactions with upstream and downstream regions within 60 kb. Next, based on the DI, a three-state hidden Markov model (HMM) was applied to determine whether a bin had interaction bias with upstream (state “U”) or downstream (“D”) regions, or no bias (“N”).

Insulator elements have not been unambiguously described before for *Arabidopsis*; if they do exist, there is no prior information on how strong their effects might be in terms of suppressing the contacts to neighboring chromatin. In addition, we did not know if the current bin size setting (2 kb) is optimal for identifying plant insulator elements. Therefore, we defined “insulator-like” regions only based on the pattern of directionality index. For type A insulator-like regions, we extracted all runs of eight HMM states “XXUUDDXX,” where “X” is any type of HMM state; we considered “UD” as insulator-like regions. Similarly, insulator regions were extracted by identifying runs of HMM states “XXUUNDDXX” (type B) and “XXUUNNDDXX” (type C); we considered “UND” and “UNND” as insulator-like regions. In total, we found 89 type A, 212 type B, and 131 type C insulator-like regions.

We defined TAD-boundary-like regions as the ends of runs of continuous “U”s or starts of runs of continuous “D”s. We used “UUUUU(D/N)(D/N)X” and “X(U/N)(U/N)DDDDD” as searching criteria, in which we considered “U(D/N)” and “(U/N)D” as TAD-boundary-like regions. In total, 540 “U(D/N)” and 818 “(U/N)D” regions were extracted. Although these two searching criteria were slightly different, there was some overlap in the results (Supplemental Fig. 31), and both partially overlapped with insulator-like regions (Supplemental Fig. 31).

On the other hand, places where a stretch of continuous “U” starts or a stretch of continuous “D” ends are analogous to the interior of TADs. Hence, “X(D/N)(D/N)UUUUU” and “DDDDD(U/N)(U/N)X” were used as search patterns, with “(D/N)U” and “D(U/N)” being considered as TAD-interior-like regions. In total, 589 “(D/N)U” and 881 “D(U/N)” partial overlapping regions were identified (Supplemental Fig. 31).

## Data access

Sequence data generated for this study have been submitted to the NCBI Sequence Read Archive (SRA; http://www.ncbi.nlm.nih.gov/sra) under accession number SRP032990.

## Supplementary Material

Supplemental Material
