# Supplementary material for: Genome-wide analysis of local chromatin packing in Arabidopsis thaliana
Source: Genome Res. 2015 Feb;25(2):246–56. doi: 10.1101/gr.170332.113 (PMC4315298; doi:10.1101/gr.170332.113)
Supplement: Supplemental Material [file supp_25_2_246__index.html]

Genome-wide analysis of local chromatin packing in Arabidopsis thaliana — Genome-wide analysis of local chromatin packing in Arabidopsis thaliana — Supplemental Material 

# Genome-wide analysis of local chromatin packing in *Arabidopsis thaliana*

## Supplemental Material

**Files in this Data Supplement:**

- Supp Table S1.xlsx
- Supp Table S2.xlsx
- Supp Table S3.docx
- Supp Table S4.txt
- Supp Table S5.txt
- Supp Table S6.txt
- Supp Table S6.txt
- Supp Table S8.txt
- Supp Table S9.txt
- Supp Table S10.xlsx
- Supplemental Material
