# Supplementary material for: Genome-wide analysis of local chromatin packing in Arabidopsis thaliana
Source: Genome Res. 2015 Feb;25(2):246–56. doi: 10.1101/gr.170332.113 (PMC4315298; doi:10.1101/gr.170332.113)
Supplement: Supplemental Material [file supp_gr.170332.113_Supp_Table_S3.docx]

Datasets used for the integration of *Arabidopsis thaliana* epigenome

|  |  |  |  |
| --- | --- | --- | --- |
| **Epigenetic Mark** | **Plant Tissue** | **Growth Condition** | **Reference** |
|  |  |  |  |
| H3K4me2 | Col-0, 2-week-old seedling | Constant light; solid half strength MS | ([Luo et al. 2012](#_ENREF_2)) |
| H3K4me3 |  |  |  |
| H3K9ac |  |  |  |
| H3K9me2 |  |  |  |
| H3K18ac |  |  |  |
| H3K27me1 |  |  |  |
| H3K27me3 |  |  |  |
| H3K36me2 |  |  |  |
| H3K36me3 |  |  |  |
| H2Bub | Col-0, 10-day-old seedling | LD, liquid half strength MS | ([Roudier et al. 2011](#_ENREF_4)) |
| H3.1 | Transgenic plants in Col-0 background, 10-day-old seedling | LD, solid MS | ([Stroud et al. 2012](#_ENREF_5)) |
| H3.3 |  |  |  |
| H3K4me1 | Col-0, 3-week-old seedling | Constant light, soil | ([Zhang et al. 2009](#_ENREF_6)) |
| H3K27me2 | Col-0, 14-day-old seedling | LD, soil | ([Park et al. 2012](#_ENREF_3)) |
| H4K5ac | Col-0, 10-day-old seedling | LD; solid half strength MS | ([Costas et al. 2011](#_ENREF_1)) |
| 5mC | Col-0, 2-week-old seedling | LD, solid half strength MS | This study |

**References:**

Costas C, de la Paz Sanchez M, Stroud H, Yu Y, Oliveros JC, Feng S, Benguria A, Lopez-Vidriero I, Zhang X, Solano R et al. 2011. Genome-wide mapping of Arabidopsis thaliana origins of DNA replication and their associated epigenetic marks. *Nat Struct Mol Biol* **18**: 395-400.

Luo C, Sidote DJ, Zhang Y, Kerstetter RA, Michael TP, Lam E. 2012. Integrative analysis of chromatin states in Arabidopsis identified potential regulatory mechanisms for natural antisense transcript production. *Plant J* **73**: 77-90.

Park S, Oh S, van Nocker S. 2012. Genomic and gene-level distribution of histone H3 dimethyl lysine-27 (H3K27me2) in Arabidopsis. *PLoS One* **7**: e52855.

Roudier F, Ahmed I, Berard C, Sarazin A, Mary-Huard T, Cortijo S, Bouyer D, Caillieux E, Duvernois-Berthet E, Al-Shikhley L et al. 2011. Integrative epigenomic mapping defines four main chromatin states in Arabidopsis. *EMBO J* **30**: 1928-1938.

Stroud H, Otero S, Desvoyes B, Ramirez-Parra E, Jacobsen SE, Gutierrez C. 2012. Genome-wide analysis of histone H3.1 and H3.3 variants in Arabidopsis thaliana. *Proc Natl Acad Sci* **109**: 5370-5375.

Zhang X, Bernatavichute YV, Cokus S, Pellegrini M, Jacobsen SE. 2009. Genome-wide analysis of mono-, di- and trimethylation of histone H3 lysine 4 in Arabidopsis thaliana. *Genome Biol* **10**: R62.
