# Supplementary material for: Genome-wide analysis of local chromatin packing in Arabidopsis thaliana
Source: Genome Res. 2015 Feb;25(2):246–56. doi: 10.1101/gr.170332.113 (PMC4315298; doi:10.1101/gr.170332.113)
Supplement: Supplemental Material [file supp_gr.170332.113_Supplemental_Material.docx]

# Supplemental information for

# Genome-wide analysis of local chromatin packing in *Arabidopsis thaliana*

Congmao Wang, Chang Liu, Damian Roqueiro, Dominik Grimm, Rebecca Schwab, Claude Becker, Christa Lanz, and Detlef Weigel


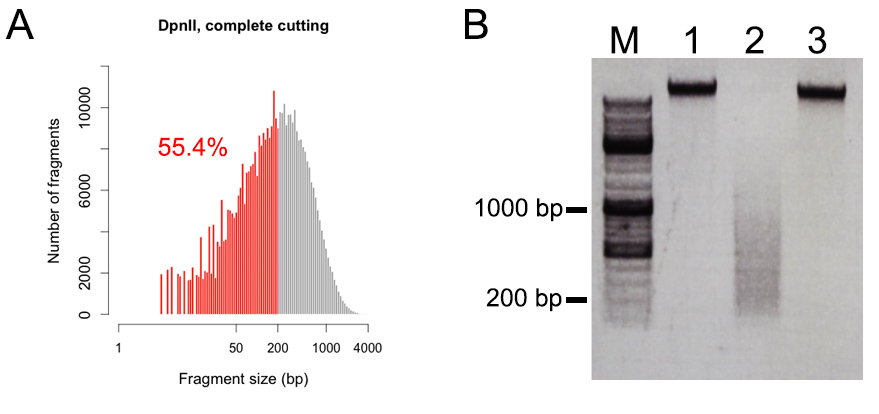


**Figure S1. Digestion of *A. thaliana* genomic DNA with four-cutter enzyme DpnII.**

(A) Predicted distribution of genomic fragments after complete DpnII digest. Products shorter than 200 bp (red) account for more than half of expected fragments. (B) Digestion of fixed chromatin. Crosslinks were reversed and DNA was purified for agarose gel separation. Lane M, marker; lane 1, intact genomic DNA; lane 2, DNA after DpnII digestion; lane 3, control sample for DNA degradation, which was treated the same as sample in lane 2 but without addition of DpnII.


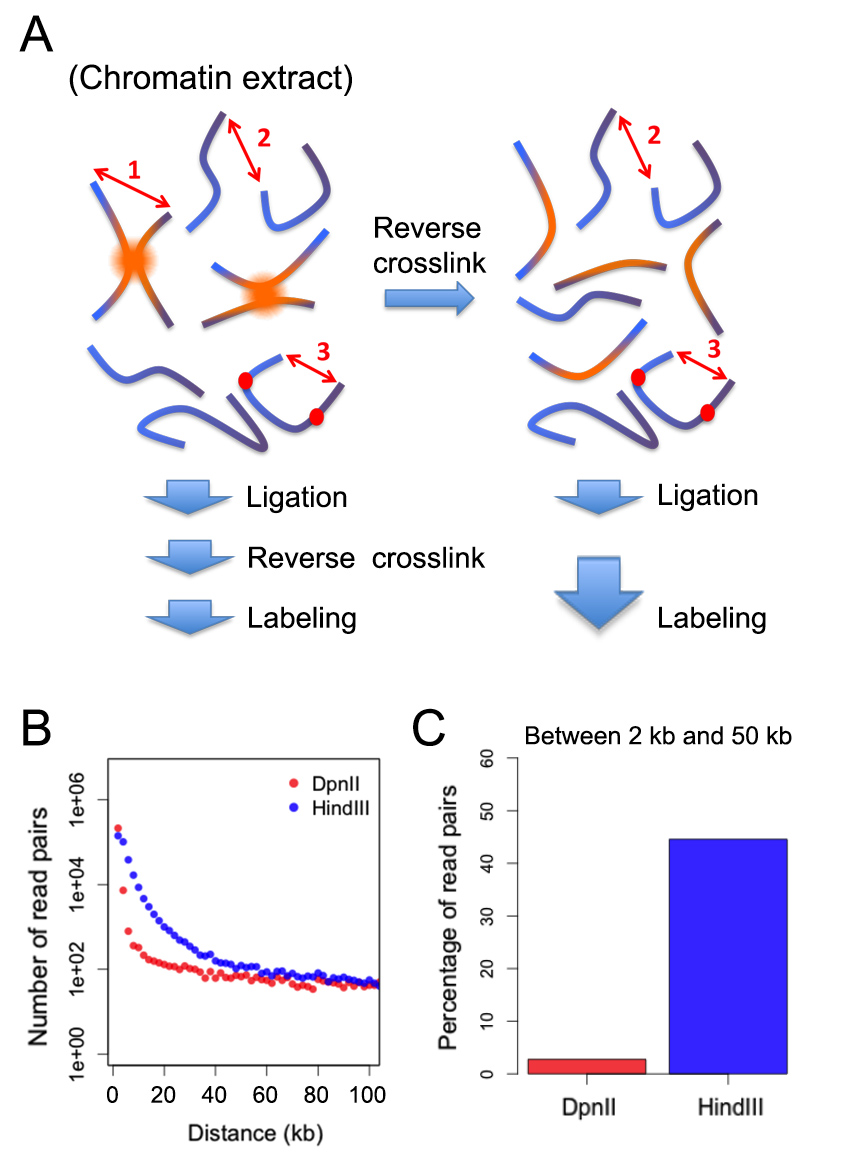


**Figure S2. Hi-C control.**

(A) Schematic diagram showing types of products anticipated from both Hi-C and control reactions. Ligation products of desired events (labeled with “1”) are only present in the Hi-C sample (left), while ligation products due to random collision between molecules (labeled with “2”) and self-ligation (labeled with “3”) are present in both Hi-C and control (right) samples. The red dots depict restriction sites in incompletely digested DNA. In the Hi-C sample, product “3” appears as a subset of product “1”. (B) Distribution of read distance in 0.55 million randomly selected control read pairs. (C) Percentage of intra-chromosomal control read pairs found within a range of 2 to 50 kb compared to the entire population of control intra-chromosomal read pairs.


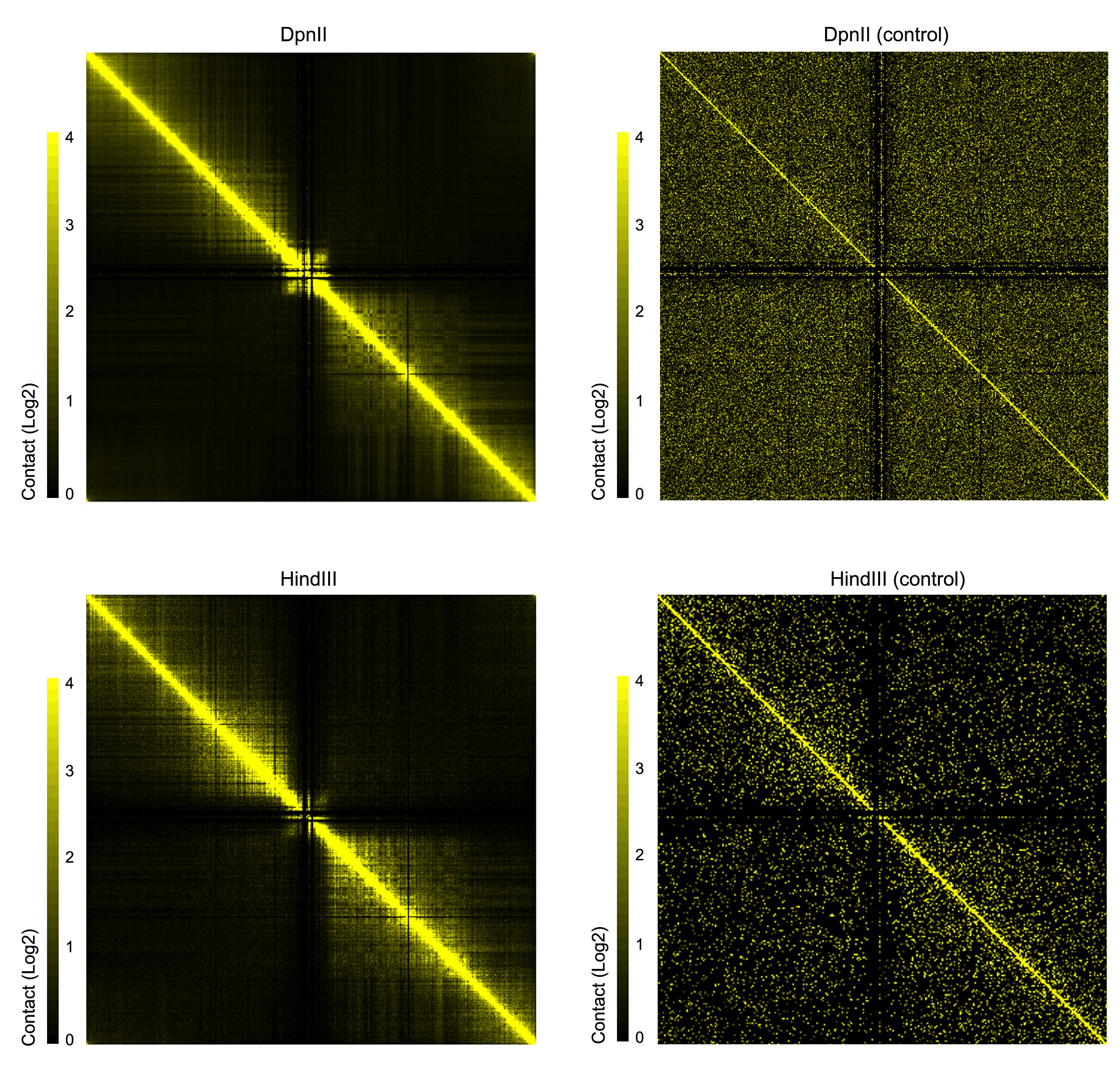


**Figure S3. Contact maps from Hi-C and control samples.**

For each sample, intra-chromosomal read pairs from chromosome 1 were selected and normalized at 20 kb resolution.


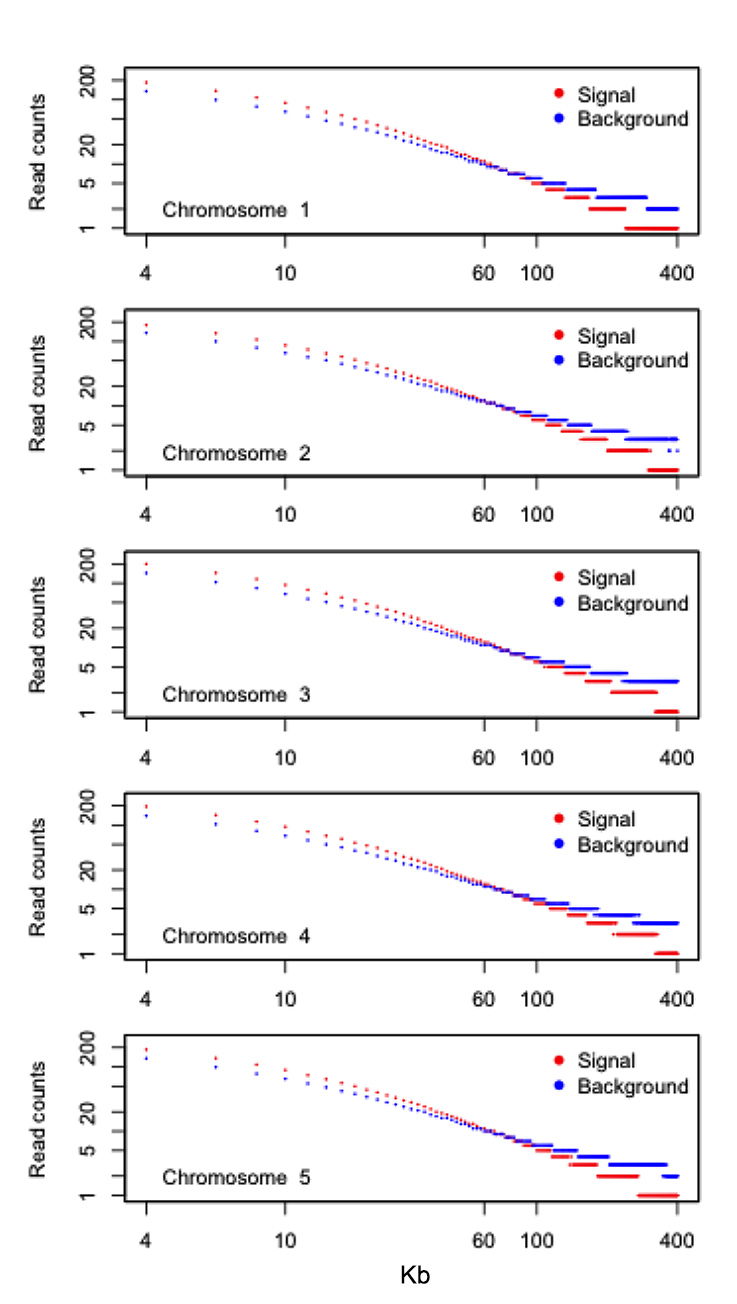


**Figure S4. Number of read pairs for interaction between 2 kb bins with different distances.**

The plots for signal and background read pairs were generated by following methods described by Hou *et al.* (Molecular Cell 48: 471-484, 2012). For each chromosome, the distances with reliable interactions are those having signal reads more than background reads.


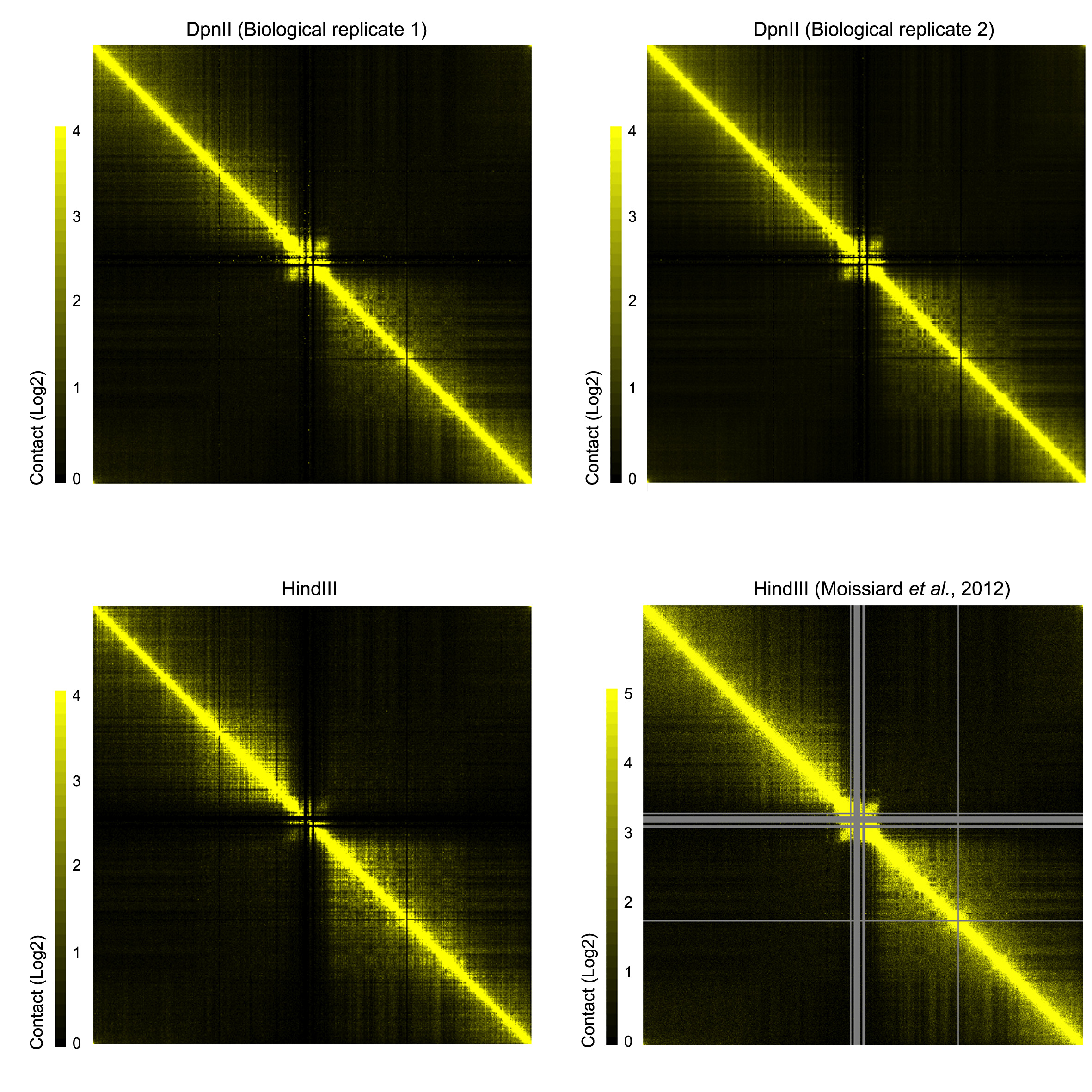


**Figure S5. Comparison of Hi-C maps among different datasets.**

For each sample, intra-chromosomal read pairs from chromosome 1 were selected and normalized in 20 kb bins. The normalized Hi-C map (at 50 kb resolution) from Moissiard et al. (Science 336: 1448-1451, 2012) was downloaded from GEO (Accession number: GSE37644).


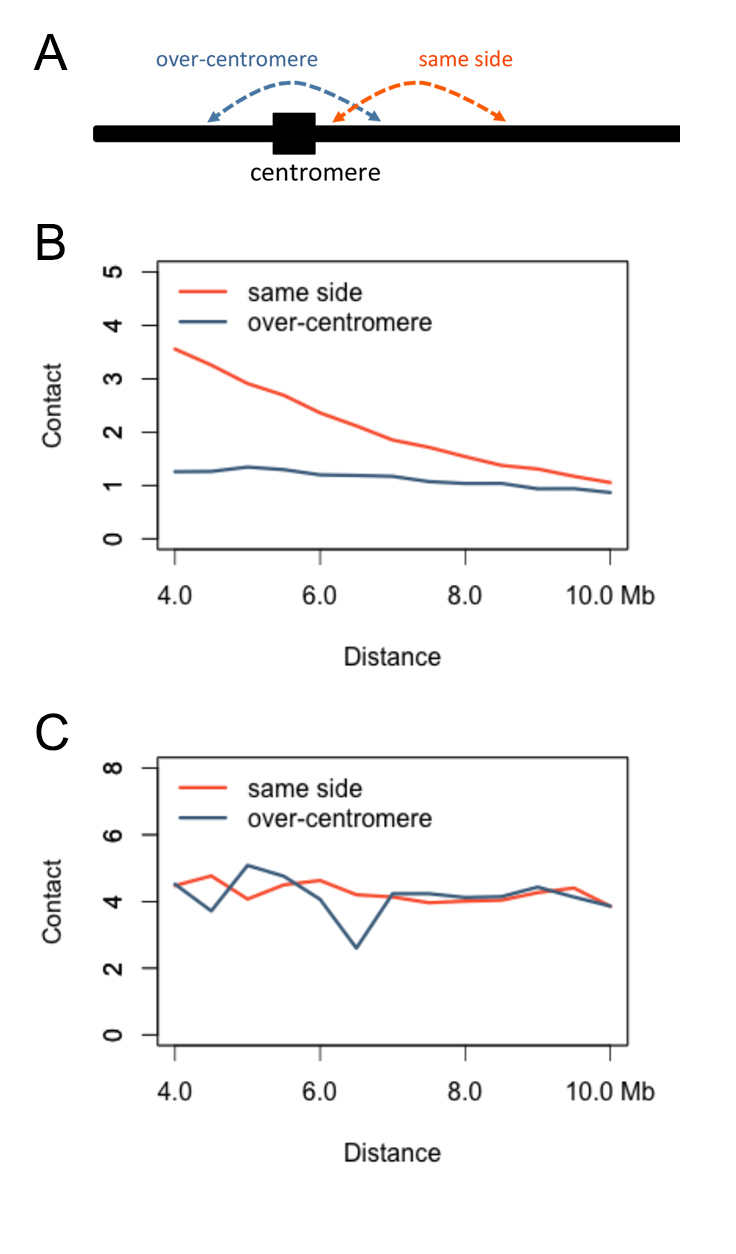


**Figure S6. Suppressed interactions across centromeres.**

(A) Schematic drawing presenting types of intra-chromosomal interactions. (B,C) Average contact strength of different intra-chromosomal interactions observed in Hi-C (B) and control (C) samples. As the normalization was performed individually on samples, only comparisons of contact strength values within, but not between different samples are meaningful.


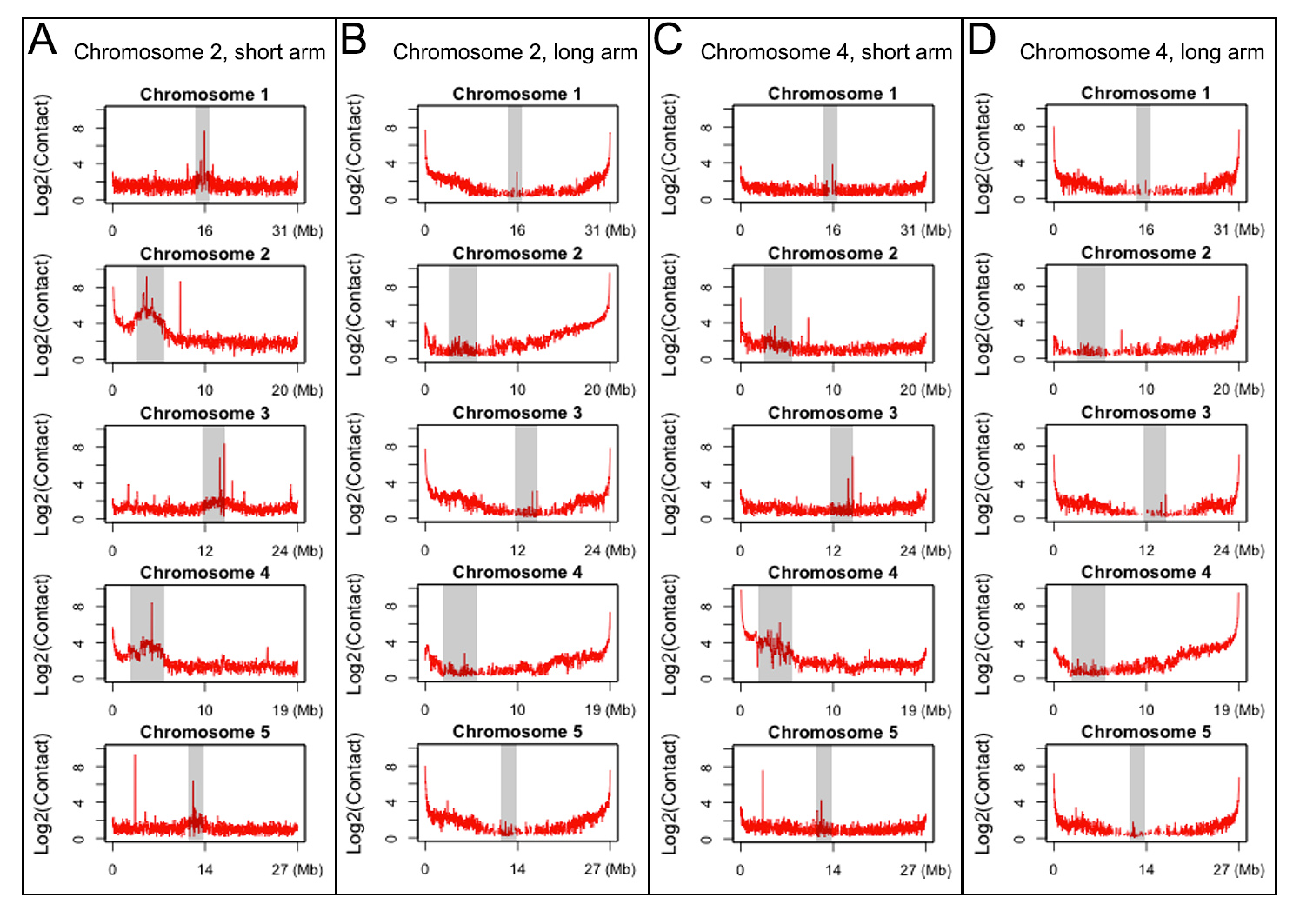


**Figure S7. Differential behaviors of telomeres.**

(A,B) Interaction of the telomeres from the short (A) and long (B) arms of chromo­some 2 with the rest of the genome. (C,D) Interaction of the telo­meres from the short (C) and long (D) arms of chromosome 4 with the rest of the ge­nome. Calculations were performed with a Hi-C map normalized at 20 kb resolution. For each panel, the terminal 60 kb was used as the query region. Shaded blocks represent centromeric regions, which were not excluded from this analysis.


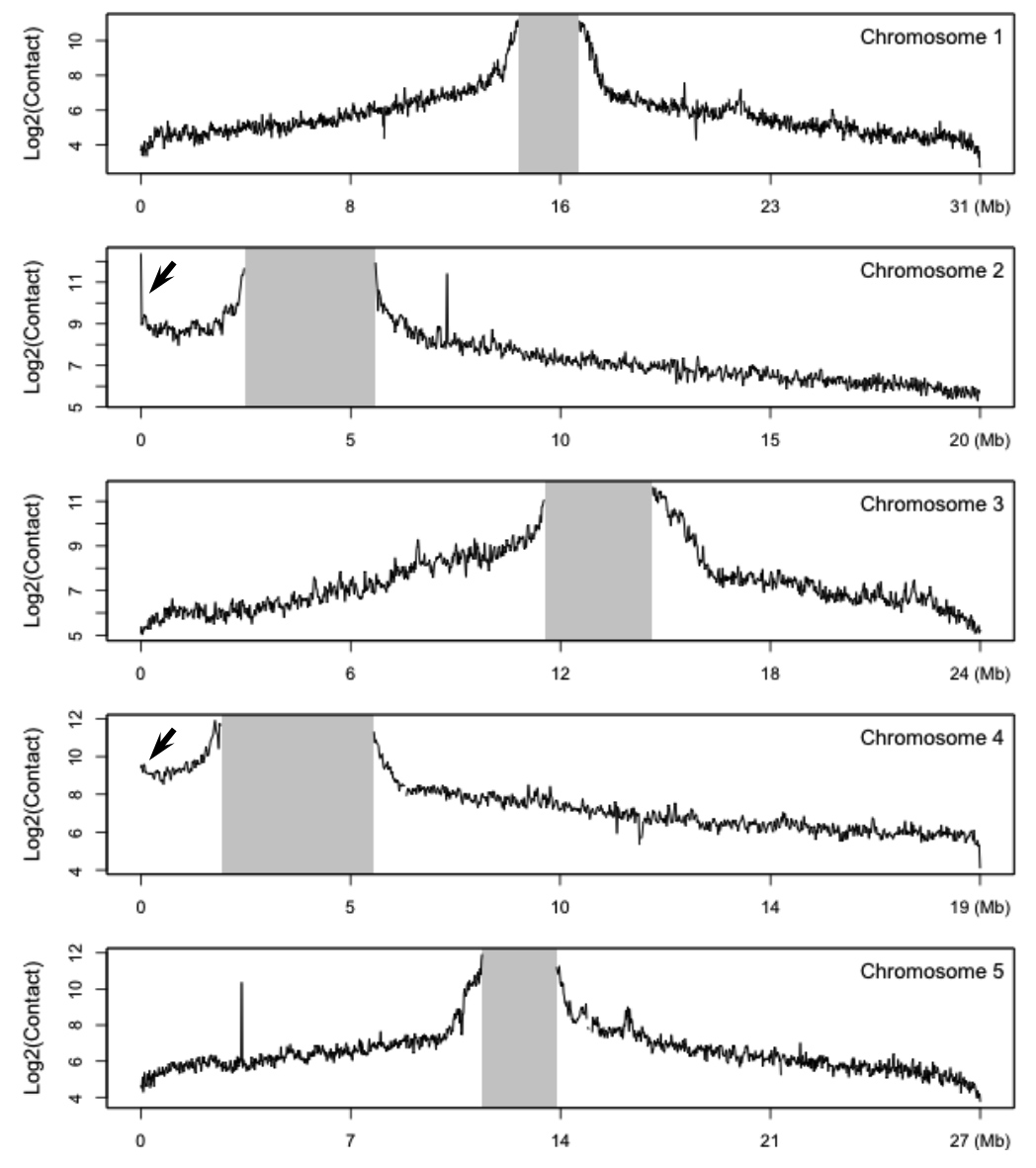


**Figure S8. Interaction between centromeric regions and telomeres.**

Calculations were performed with Hi-C map normalized at 20 kb resolution. For each panel, bins belonging to centromeres (shaded regions) were used as query. Arrows indicate stronger interactions found with telomeres of the short arms of chromosomes 2 and 4.


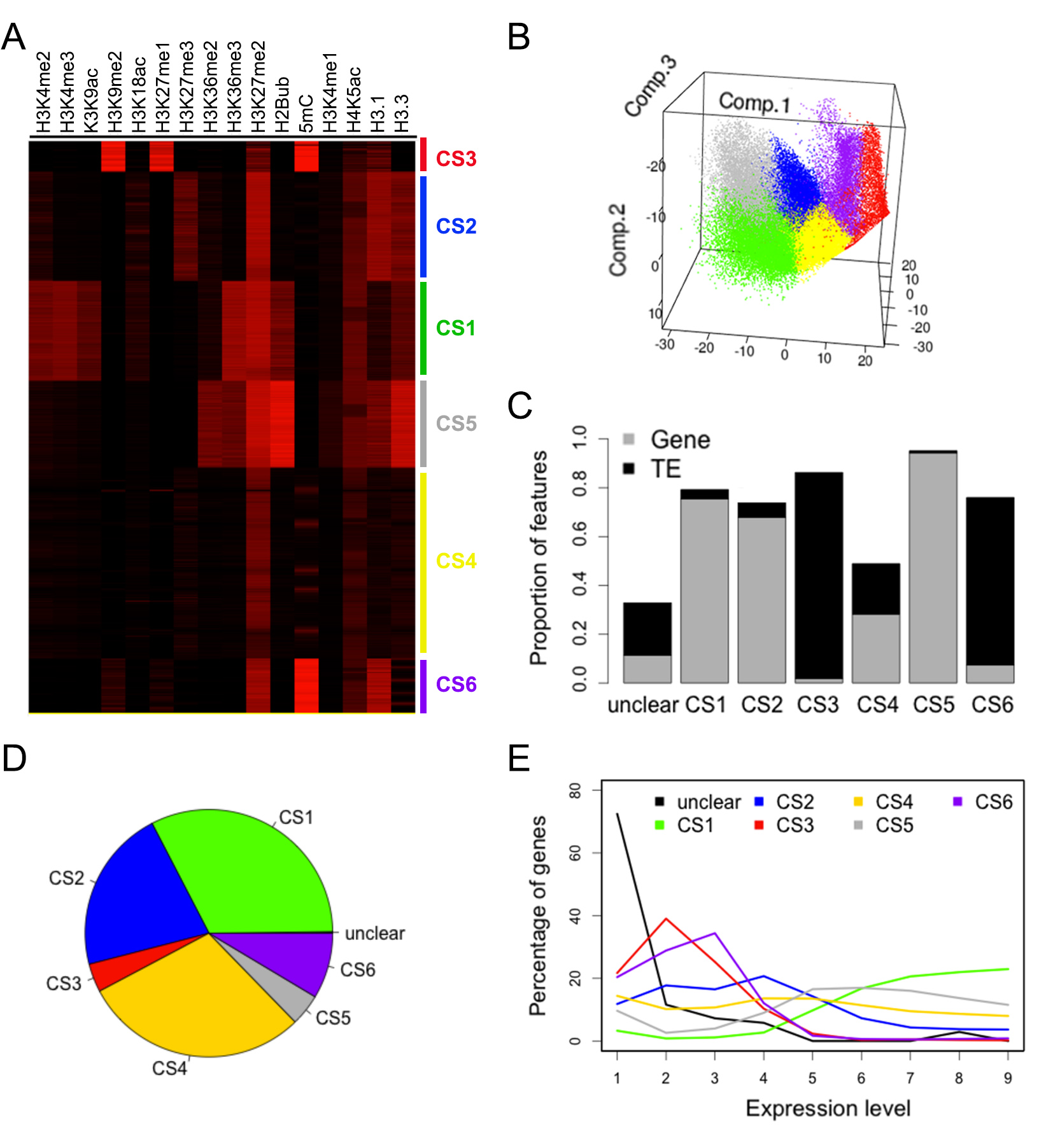


**Figure S9. Classification of the *A. thaliana* epigenome at 2 kb resolution.**

(A) Identification of chromatin states (CS). CS1-4 are according to Roudier and colleagues (EMBO J 30: 1928-1938, 2011), who used 12 epigenetic data sets that partially overlap with the 16 data sets used here. CS5 (grey) is related to CS1 (green), and CS6 (purple) to CS3 (red). The naming of CS1-6 groups is the same as that for the epigenome approximated with 400 bp bins. (B) Visualization of classified bins with the first three principal components. (C) Distribution of gene bodies and TEs in CS groups. (D) Distribution of genes, based on the location of their TSSs, in each CS group. (E) Distribution of gene expression level in each CS group; see Supplemental Fig. 16B for detailed definition of expression levels.


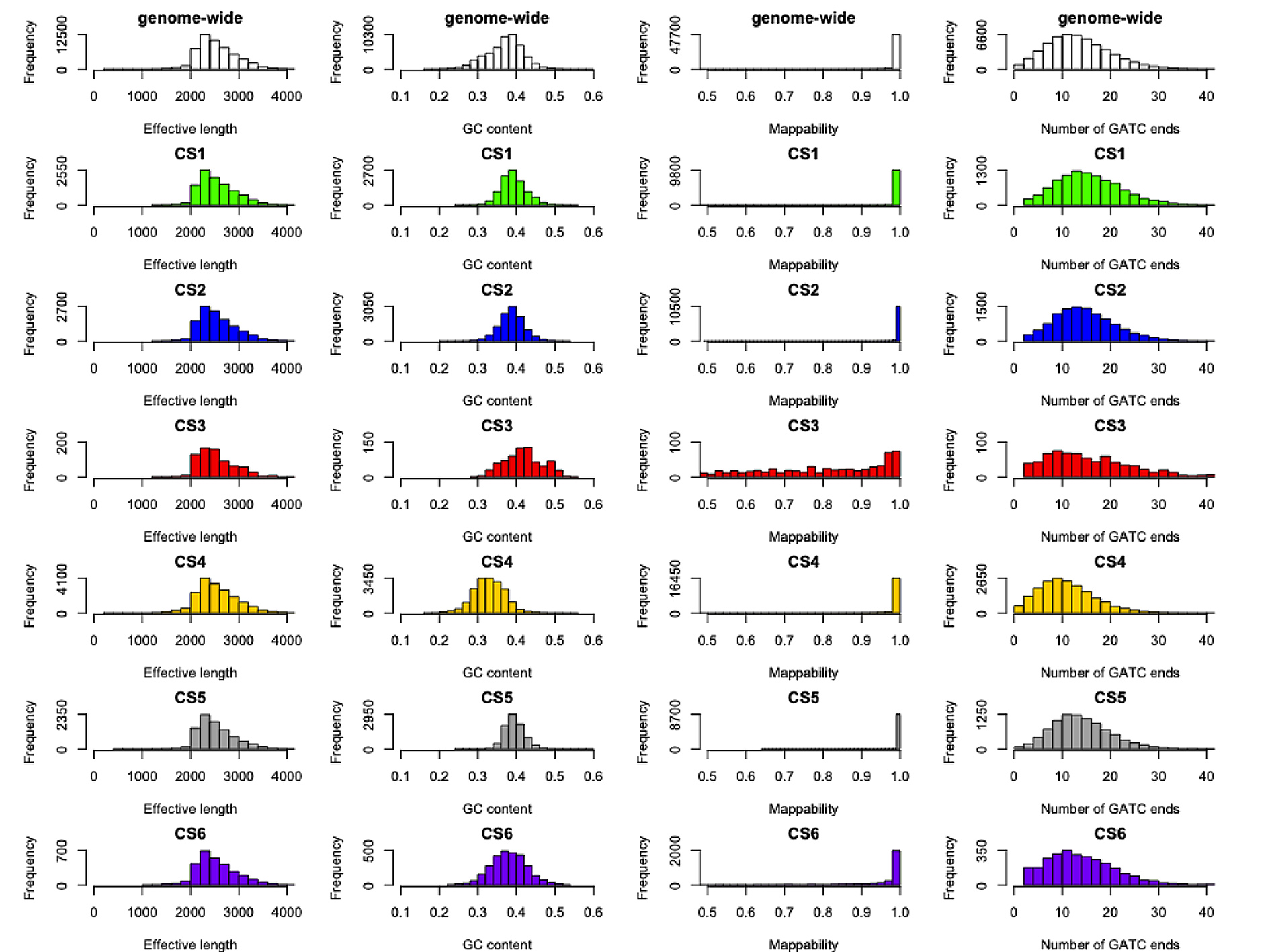


**Figure S10. Bias analysis of bins grouped according to chromatin states.**

The distribution patterns of effective length, GC content, mappability, and number of GATC ends are plotted for bins belong to each CS group. Bins not included for Hi-C map normalization and bins belonging to centromere are not included in the plots.


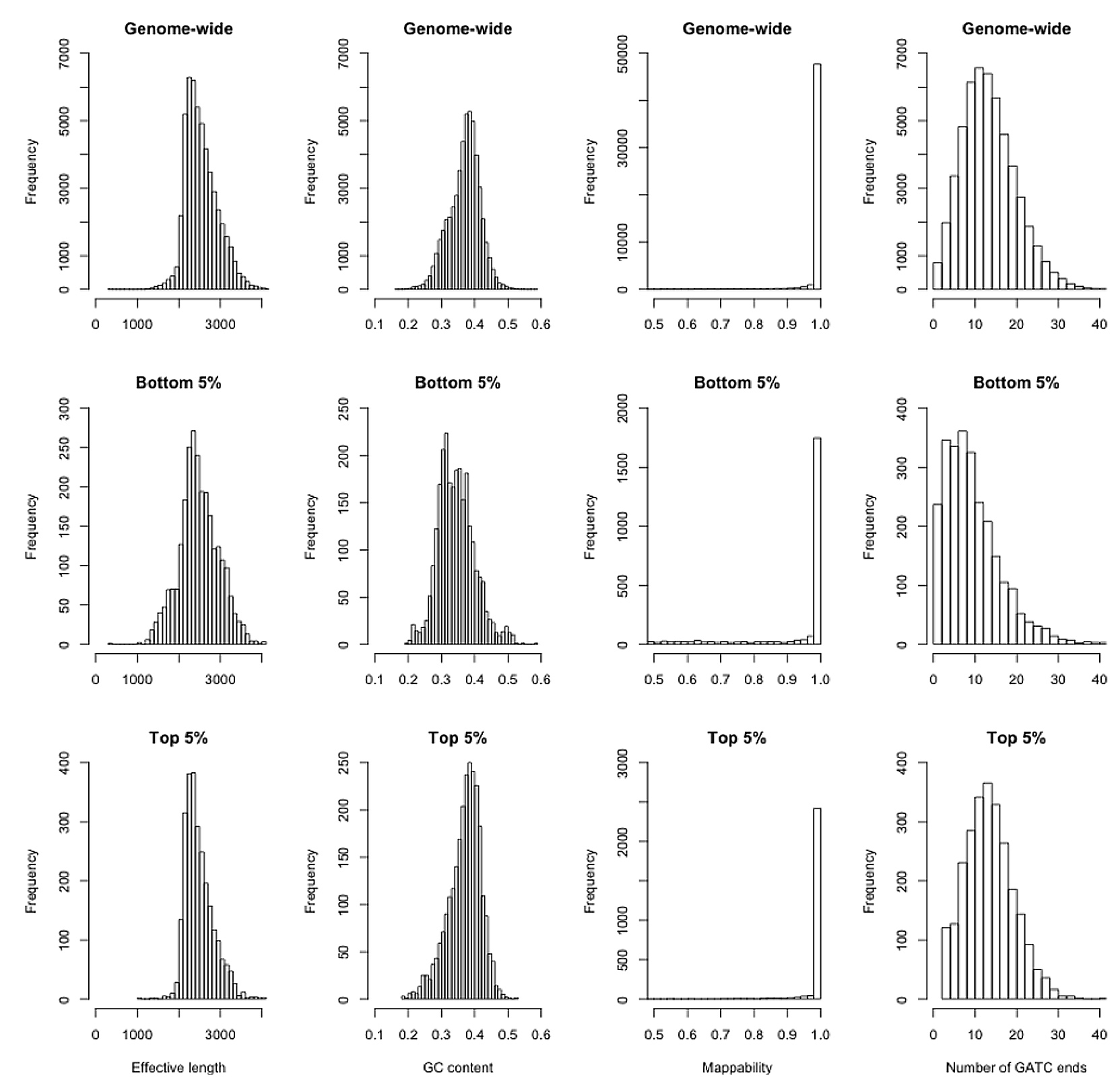


**Figure S11. Bias analysis of bins showing extreme week or strong contact strength.**

The distribution patterns of effective length, GC content, mappability, and number of GATC ends are plotted for bins with the 5% lowest or highest contact strength.


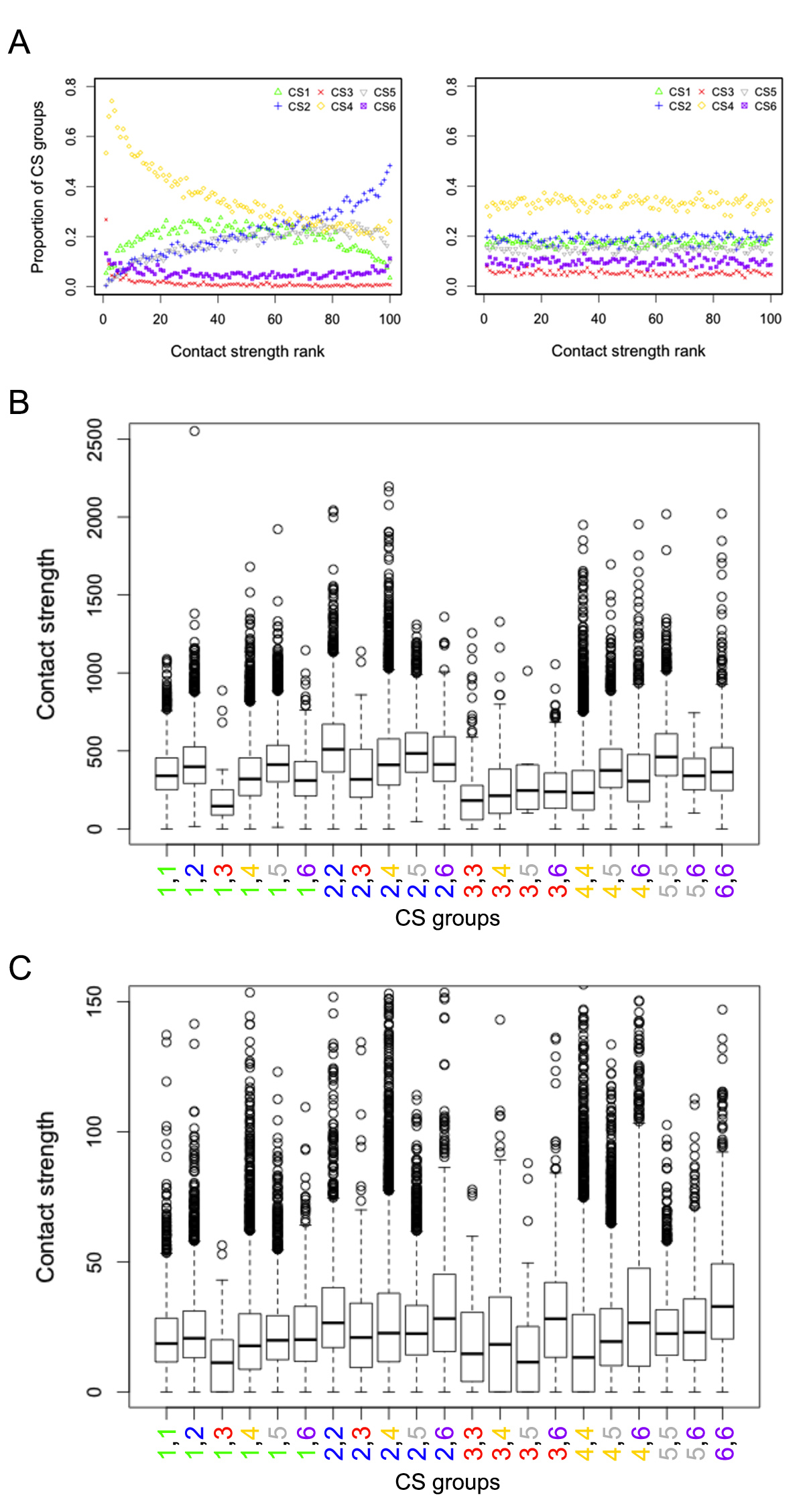


**Figure S12. Association between contact strength and epigenetic marks.**

(A) Relationship between proportion of CS groups of bins and rank of contact strength (10 kb)(left panel). Results from bins from CS1 to CS6 are all shown here, while results without CS3 and CS4 are shown in Figure 3C. Right panel, the local contact values were permuted and randomly assigned back to each bin, and bins are subsequently ranked based their new values. (B,C) Contact strength of adjacent bins (B) and bins at distance of 100 kb (C). For panel B, the plot without CS3 and CS4 is also shown in Figure 3D. Bins with unclear category are not shown since they are of small number.


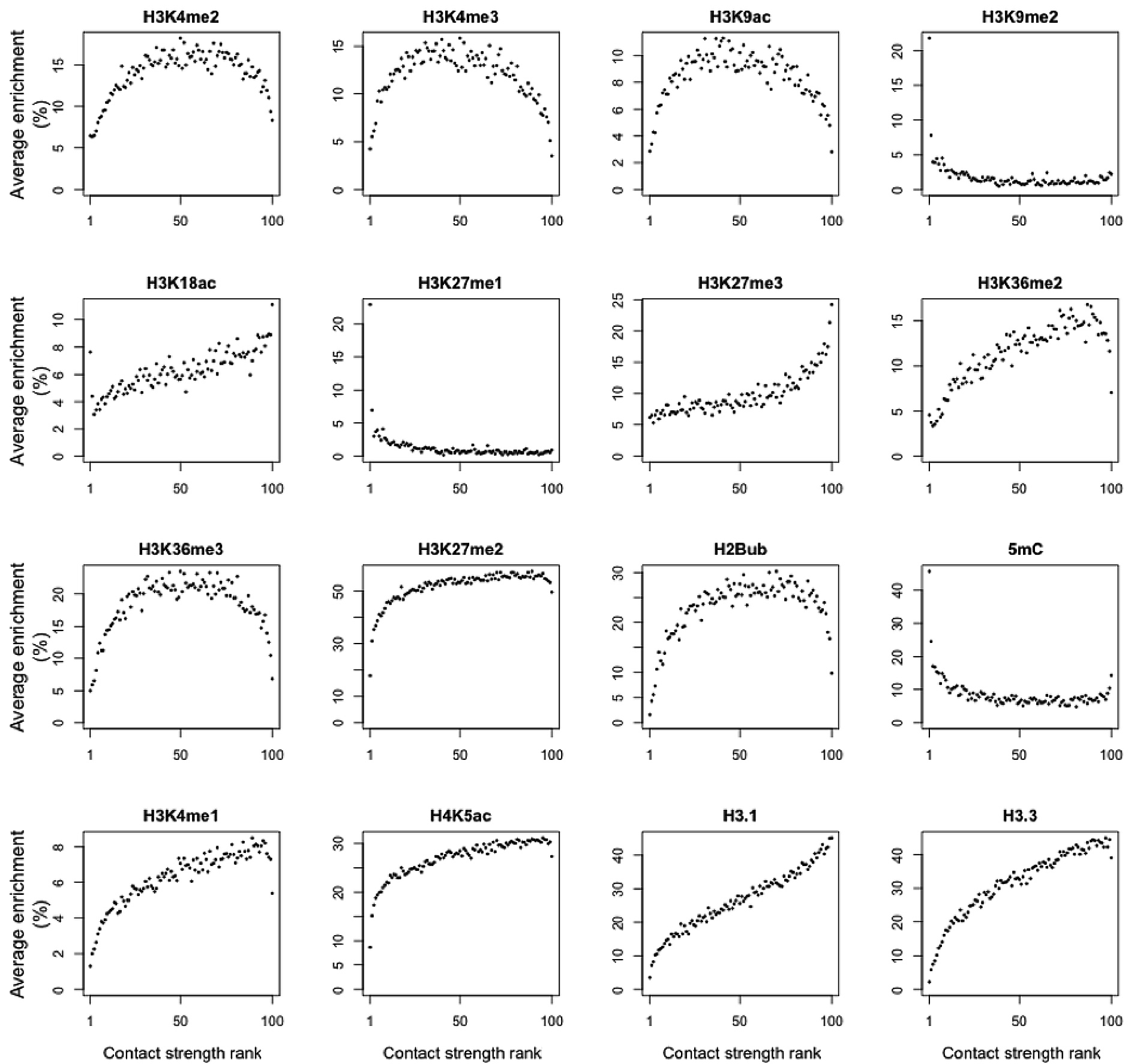


**Figure S13. Association between local contact strength of bins and epigenetic marks.**

Average enrichment of 16 epigenetic marks in bins ranked based on local contact values is plotted. Rank 1 represents bins with the lowest contact values. Y-axis indicates percentage of nucleotides in 2 kb bins.


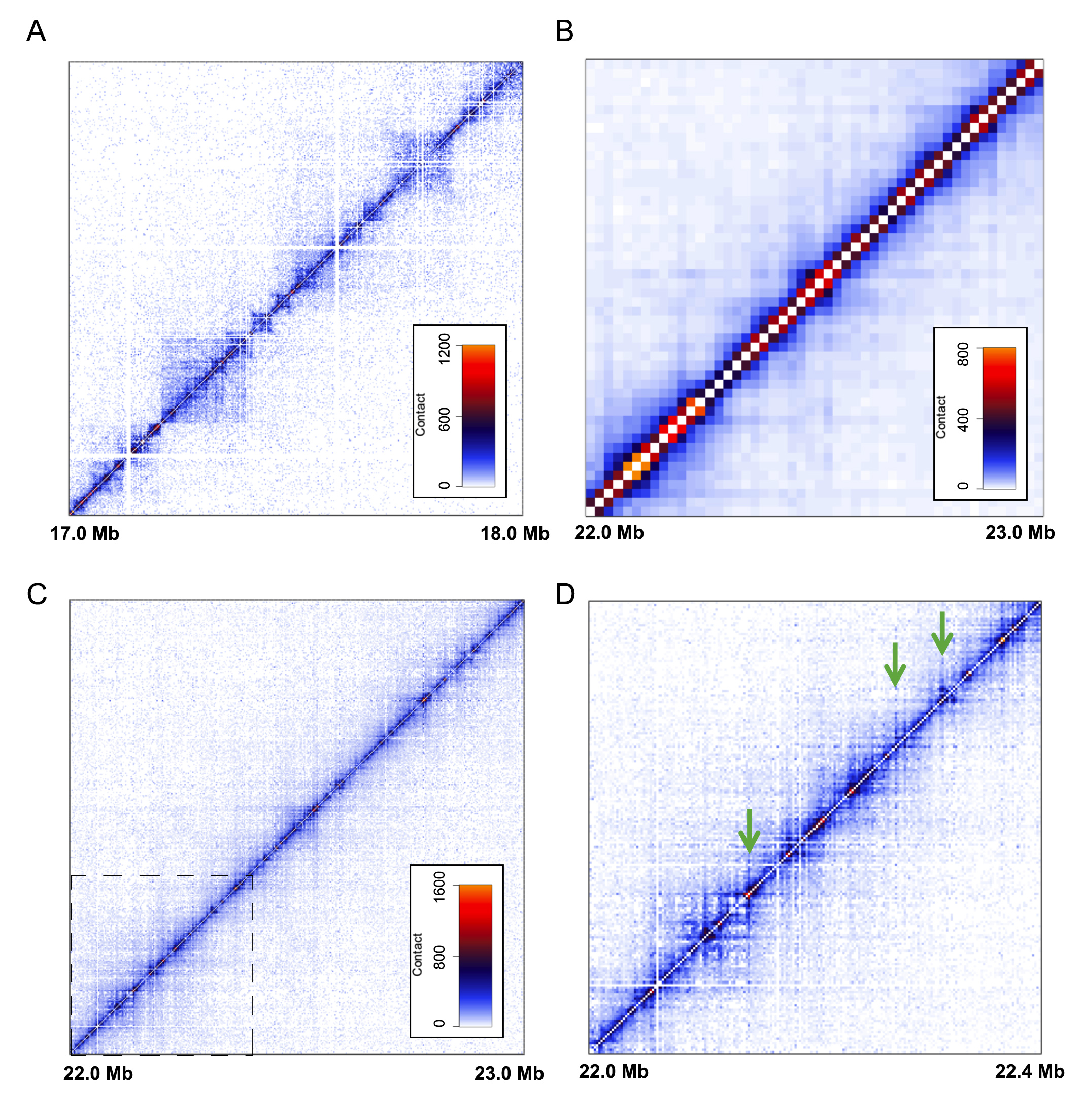


**Figure S14. The *A. thaliana* Hi-C map is characterized by local strips.**

(A) Hi-C map of a region from *Drosophila* chromosome 3R normalized at 2 kb. The raw sequencing data from Sexton *et al.* (Cell 148: 458-472, 2012) was downloaded from GEO (Accession number: GSE34453). (B-D) Hi-C map of a region from *A. thaliana* chromosome 1 normalized at 20 kb (B) and 2 kb (C,D). (D) Part of the region highlighted with dash-line in (C) is shown. Green arrows depict positive strips.


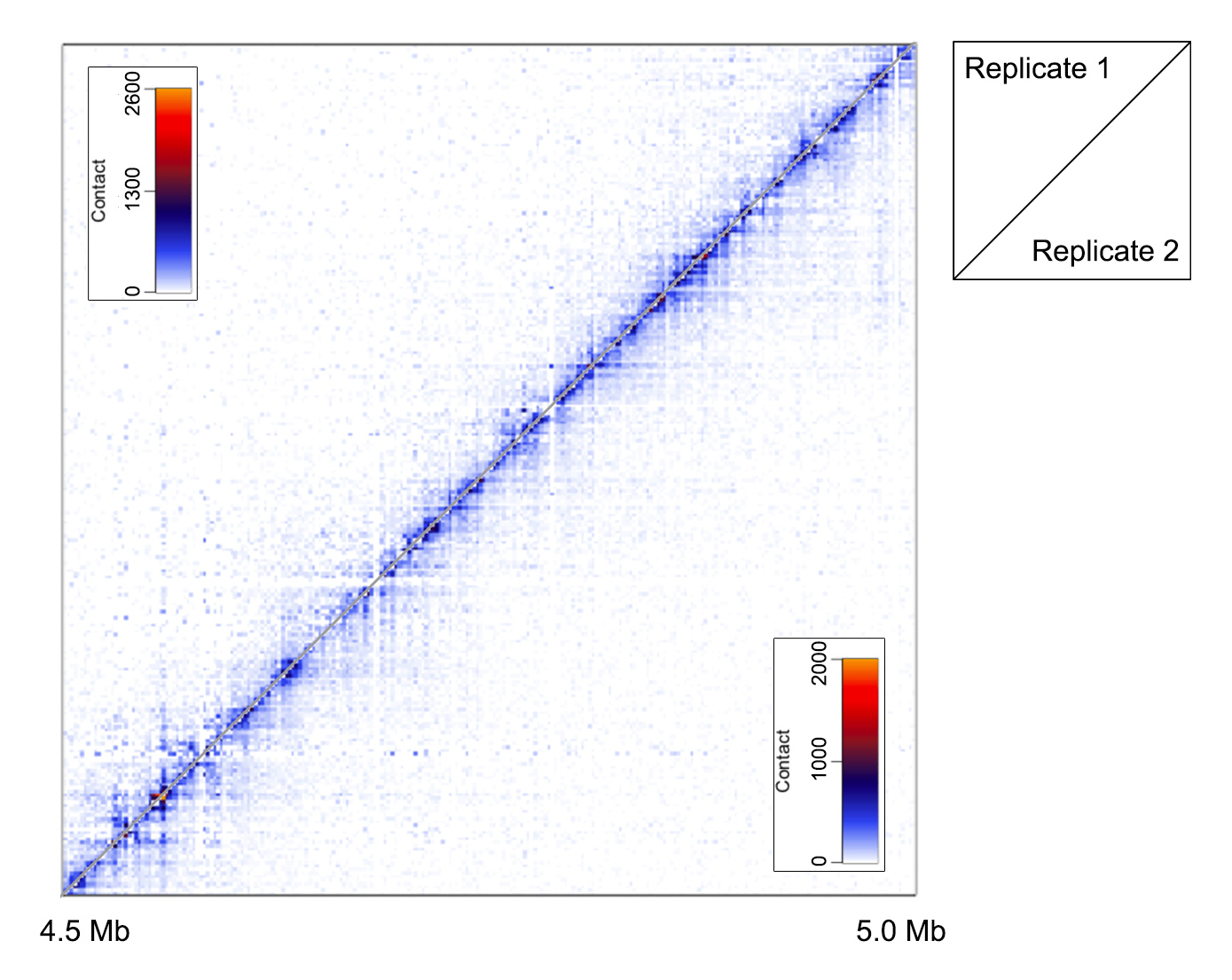


**Figure S15. Consistent detection of local strips in biological replicates.**

A region from chromosome 1 is shown.


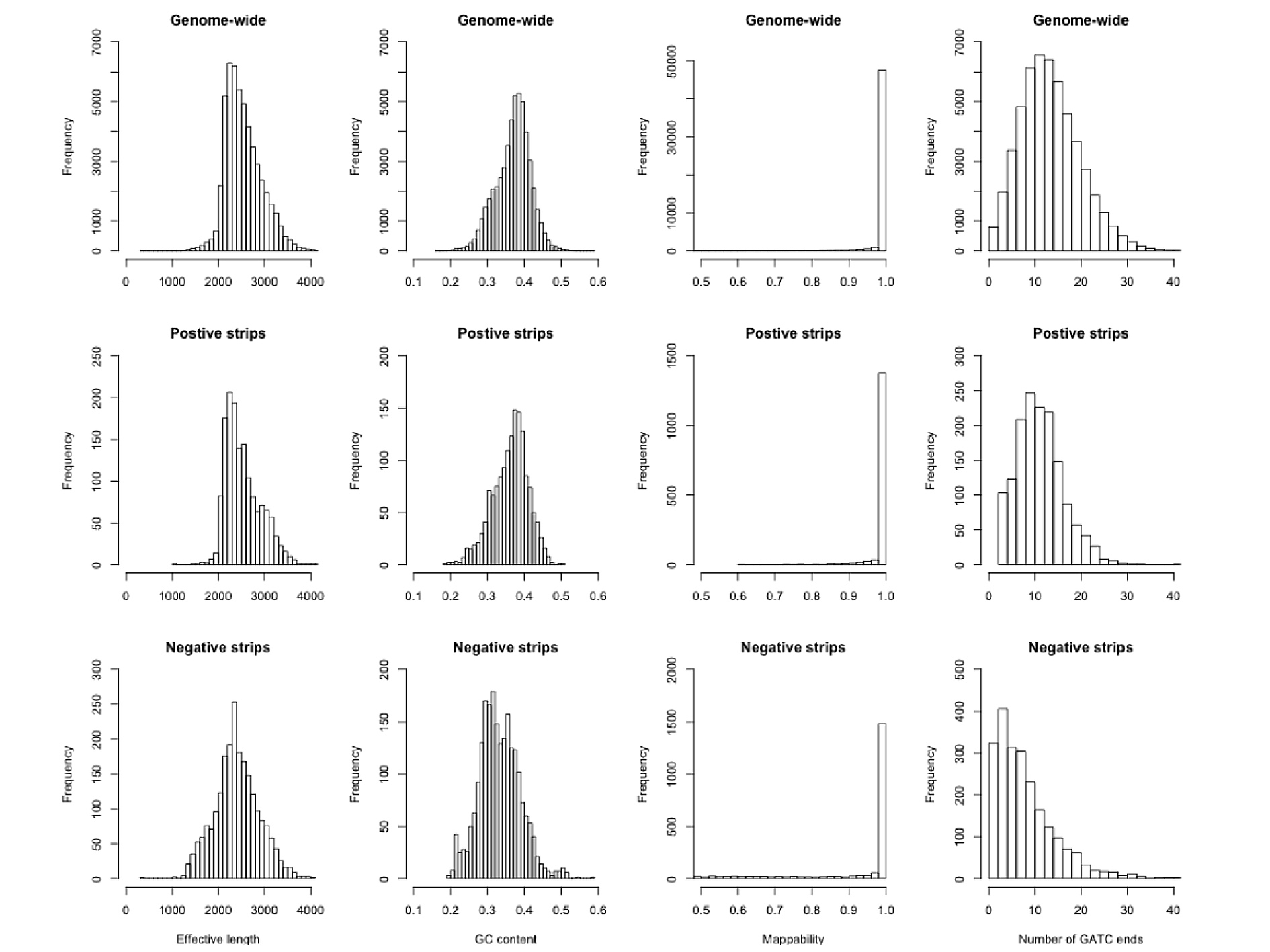


**Figure S16. Bias analysis of bins forming strips on Hi-C map.**

The distribution patterns of effective length, GC content, mappability, and number of GATC ends are plotted for bins showing positive and negative strips, respectively.


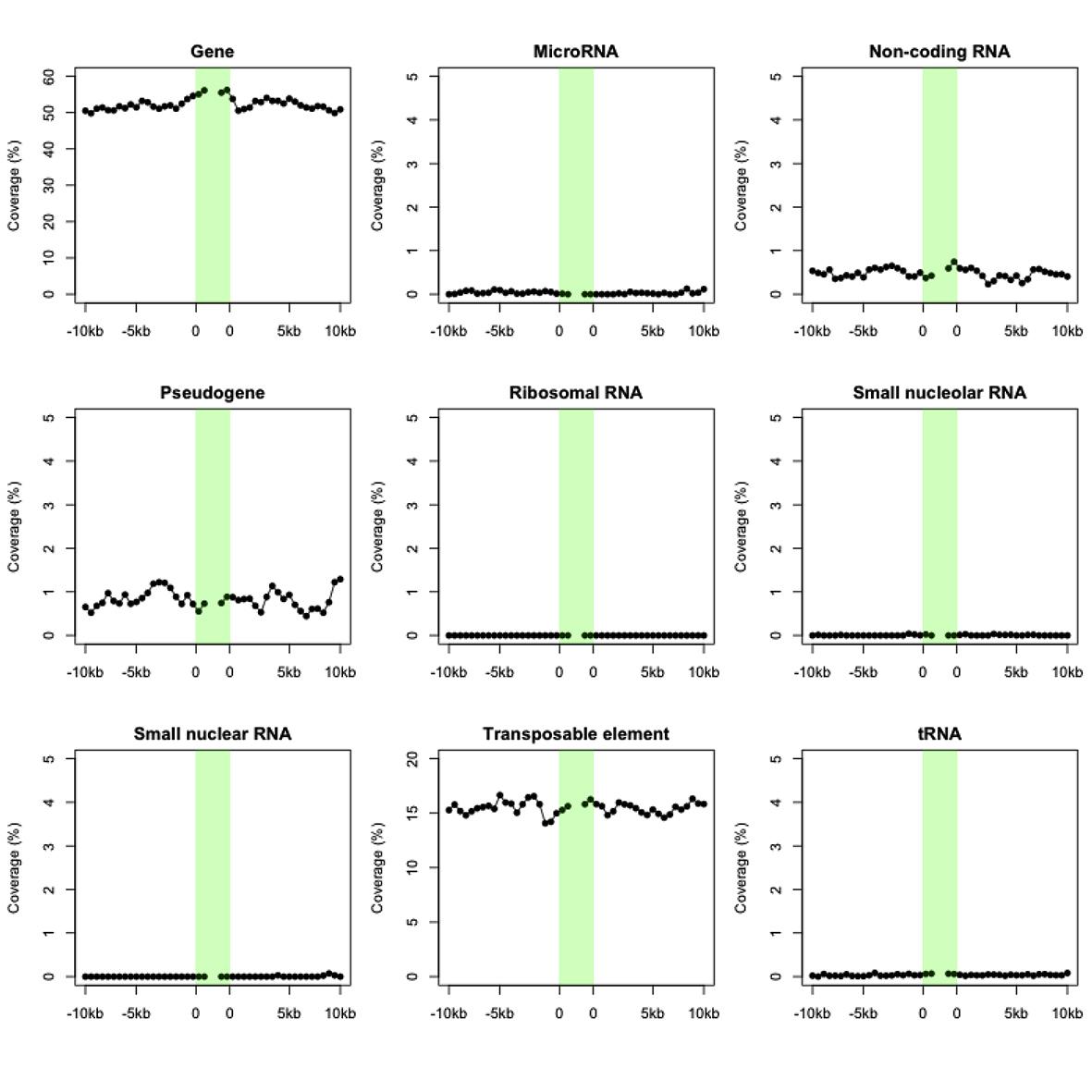


**Figure S17. Genomic features around positive strips.**

Various genomic features around positive strips. Coverage means the average percentage of each 500 bp bin annotated with the respective sequence feature.


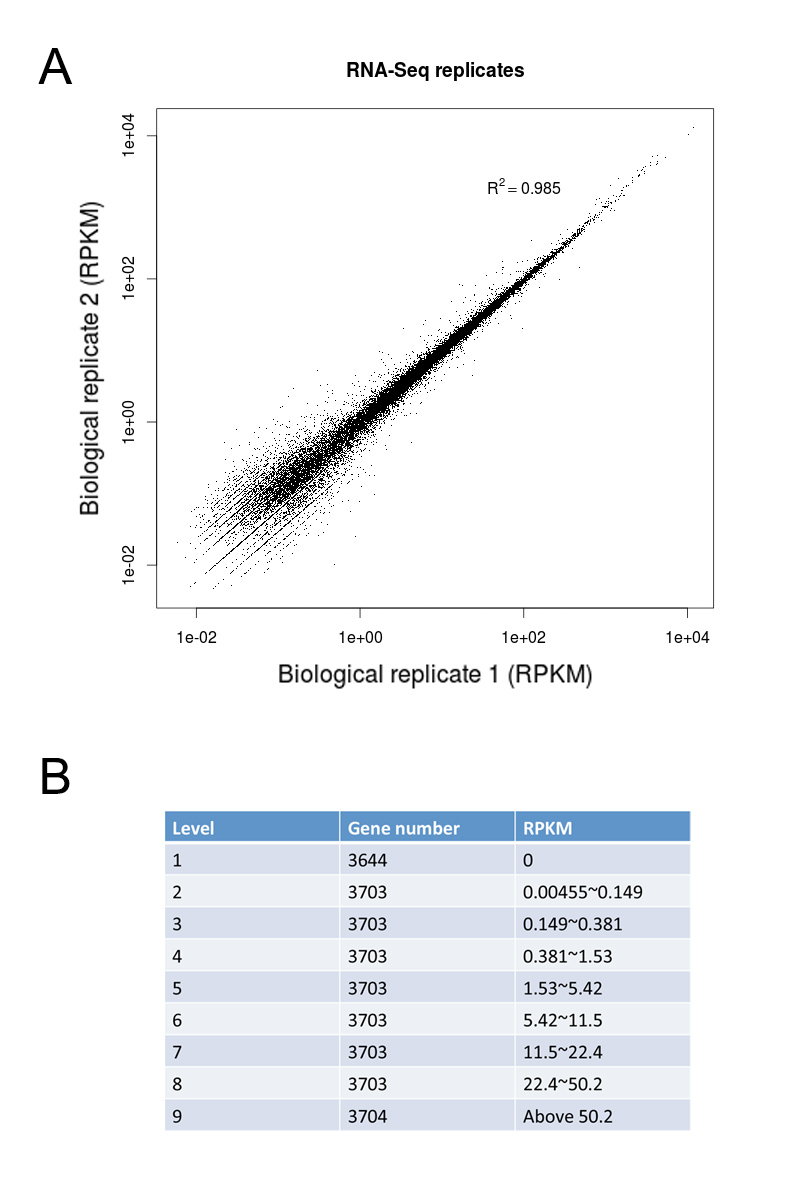


**Figure S18. Categorization of gene expression levels.**

(A) Comparison of RNA-seq data generated from two biological replicates. (B) Grouping genes into 9 expression levels according to their RPKM values. Each group has a similar number of genes.


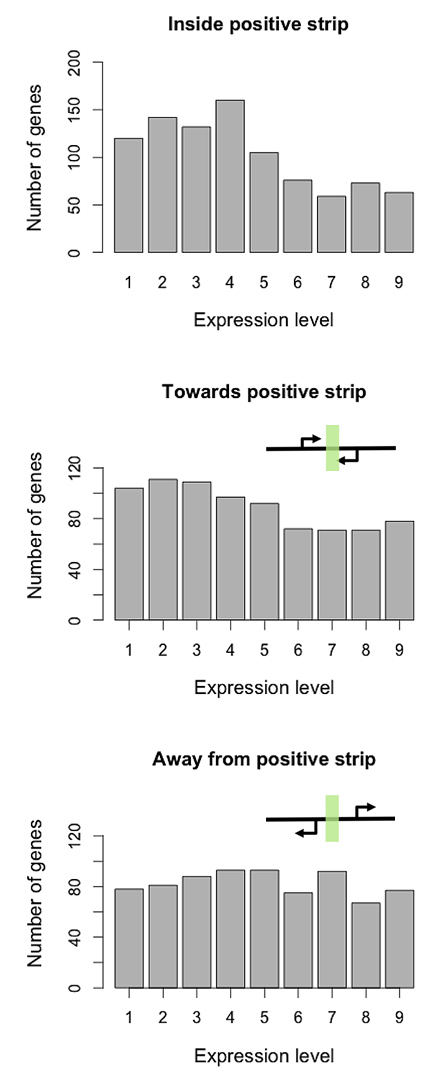


**Figure S19. Gene expression levels and positive strips.**

Distribution of genes by expression level around positive strips were plotted. For genes outside strips, only those with TSS located within 2 kb from strip borders were included, and they were further grouped by their transcription directions with respect to strips.


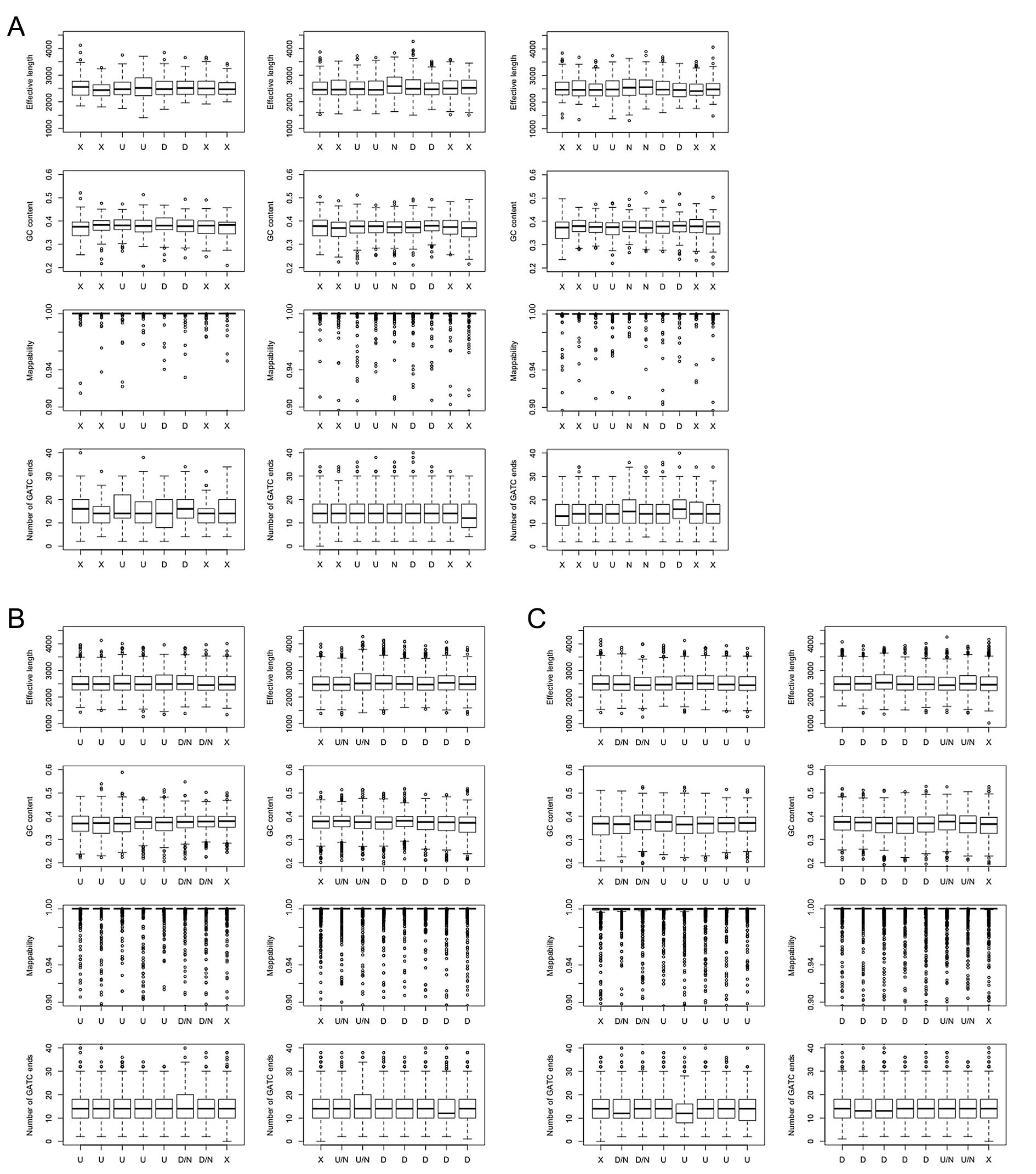


**Figure S20. Bias analysis of bins forming specific sequence of HMM states.**

Distribution of effective length, GC content, mappability, and number of GATC ends (DpnII recognition sequence) are plotted for bins around insulator-like regions (A), TAD-boundary-like regions (B), and TAD-interior-like regions (C). “U” and “D” mean bins having biased interaction with upstream and downstream regions. “N” means bins without directionality bias. “X” means bins with any HMM state.


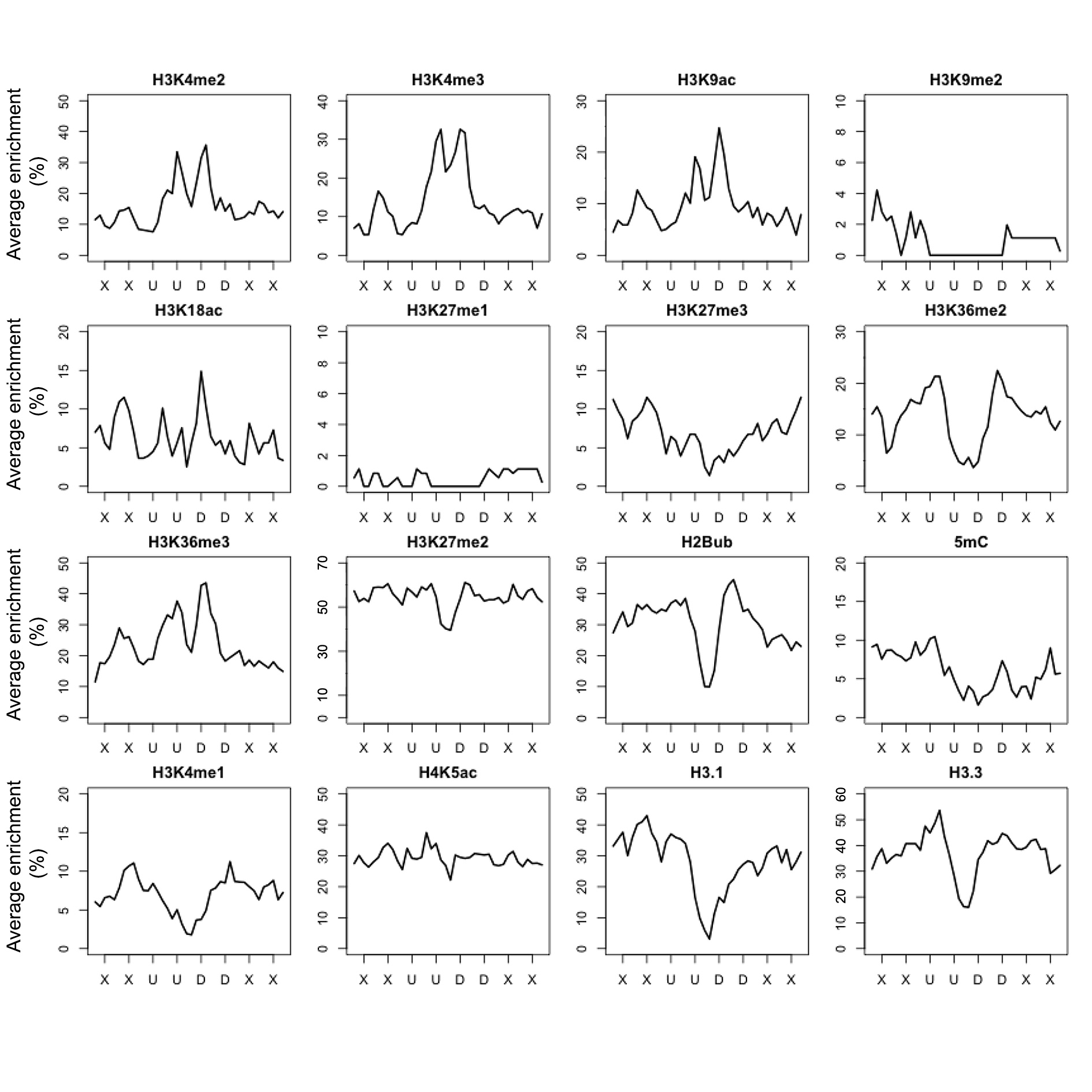


**Figure S21. Epigenetic marks associated with type A insulator regions.**

Average enrichment refers to fraction of each 400 bp region identified as enriched for the respective epigenetic mark. “U” and “D” mean bins having biased interaction with upstream and downstream regions, respectively. “X” means bins with any HMM state.


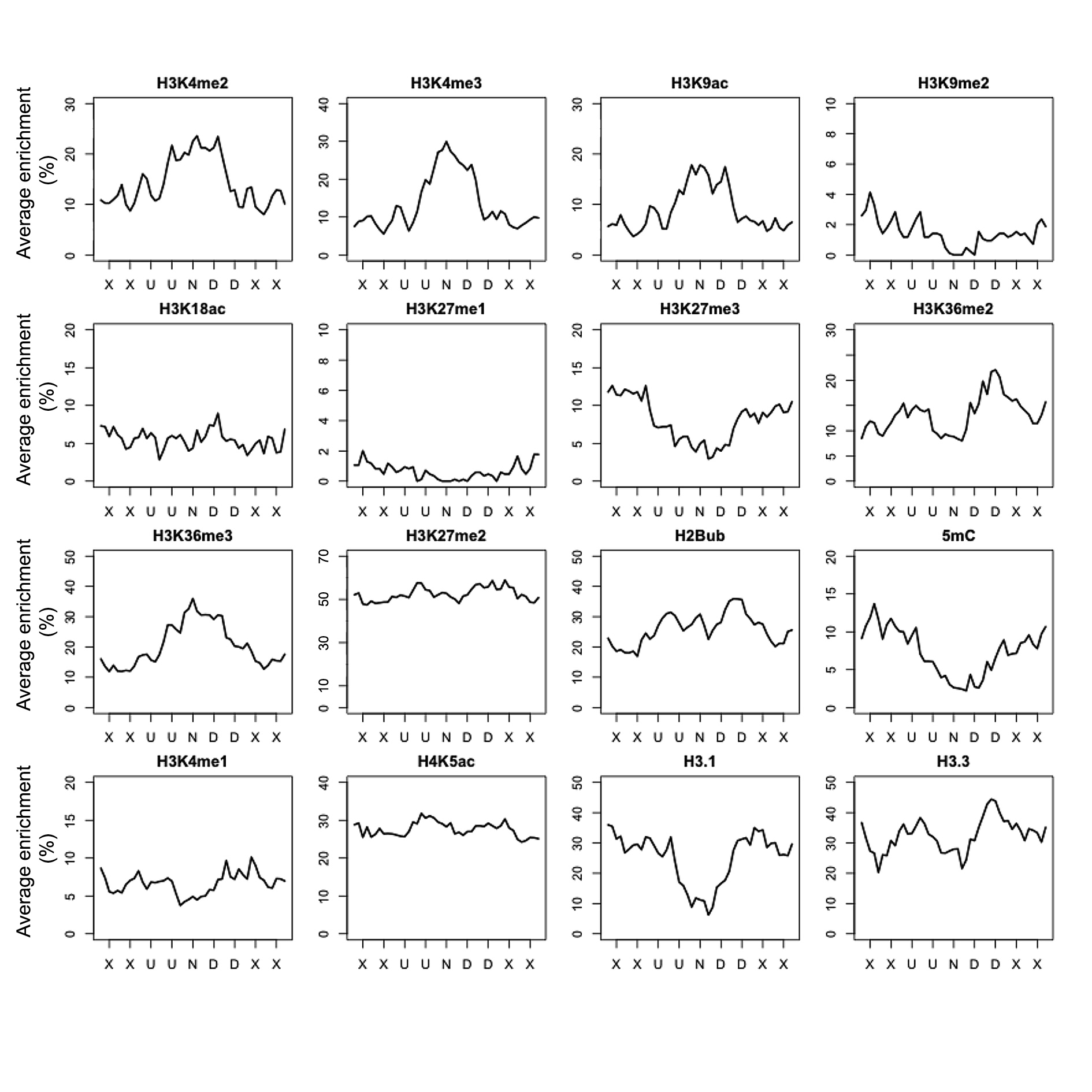


**Figure S22. Epigenetic marks associated with type B insulator regions.**

Average enrichment refers to fraction of each 400 bp region identified as enriched for the respective epigenetic mark. “U” and “D” mean bins having biased interaction with upstream and downstream regions, respectively. “N” means bins without directionality bias. “X” means bins with any HMM state.


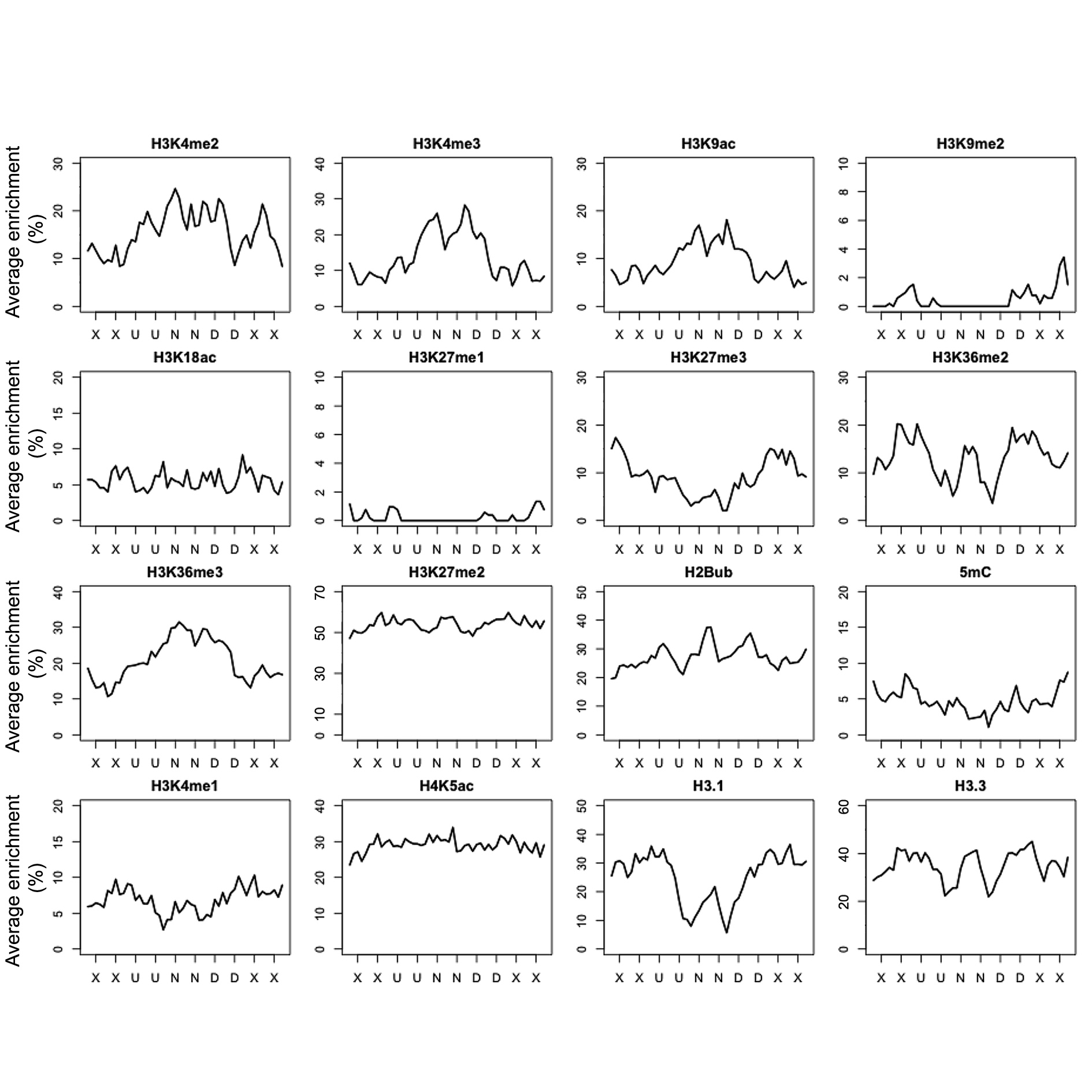


**Figure S23. Epigenetic marks associated with type C insulator regions.**

Average enrichment refers to fraction of each 400 bp region identified as enriched for the respective epigenetic mark. “U” and “D” mean bins having biased interaction with upstream and downstream regions. “N” means bins without directionality bias. “X” means bins with any HMM state.


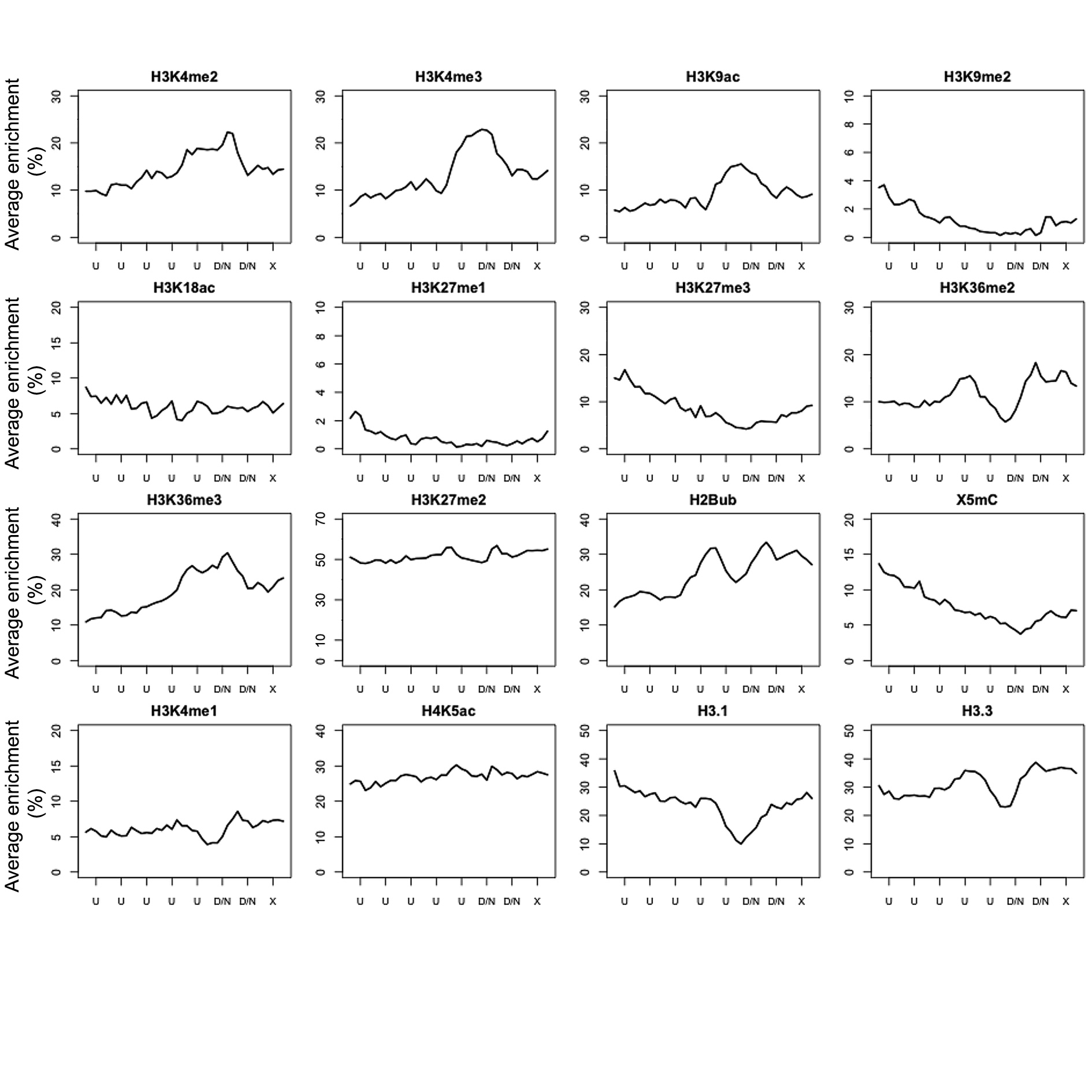


**Figure S24. Epigenetic marks associated with TAD-boundary-like regions at the end of a “U” run.**

Average enrichment refers to fraction of each 400 bp region identified as enriched for the respective epigenetic mark. “U” and “D” mean bins having biased interaction with upstream and downstream regions, respectively. “N” means bins without directionality bias. “X” means bins with any HMM state.


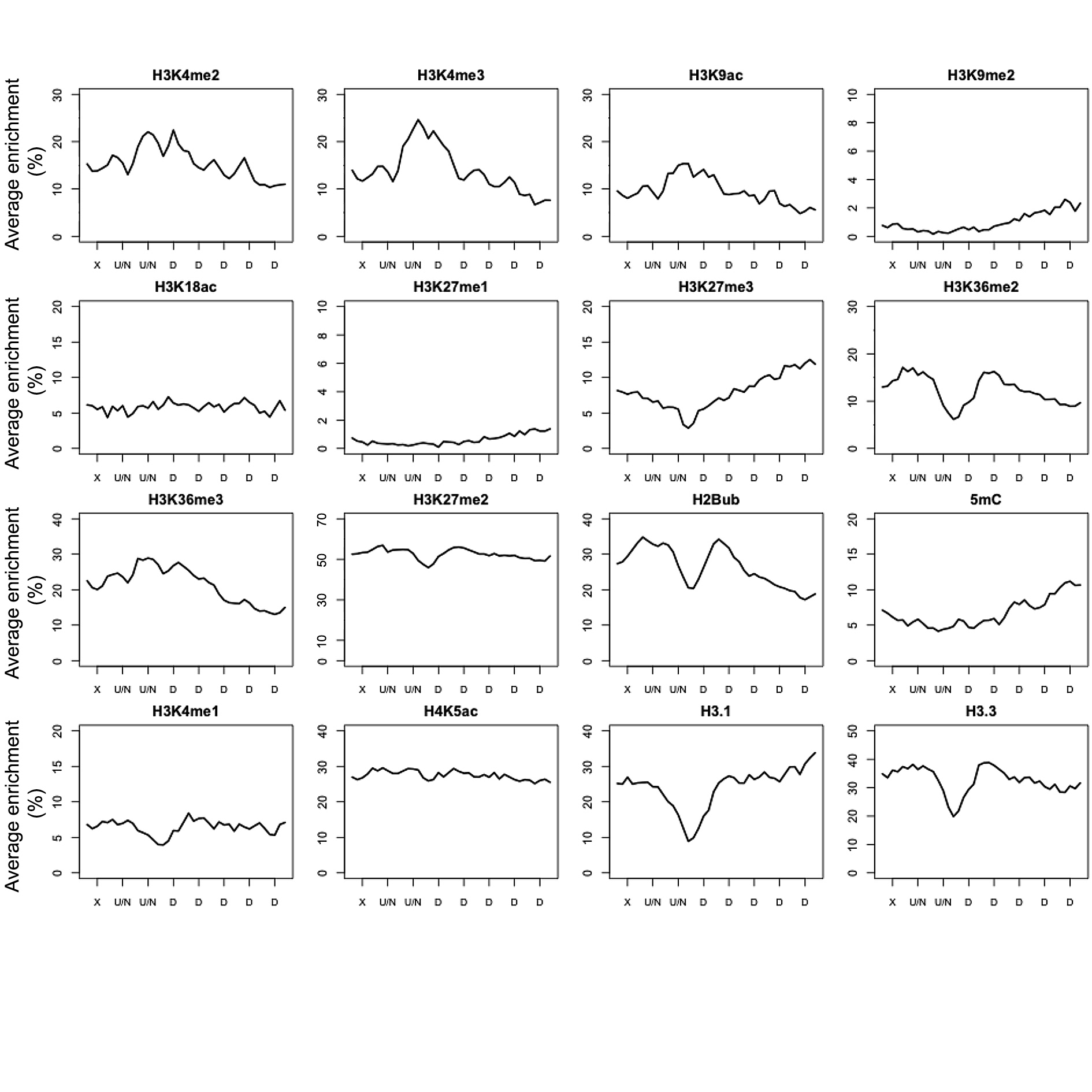


**Figure S25. Epigenetic marks associated with TAD-boundary-like regions at the start of a “D” run.**

Average enrichment refers to fraction of each 400 bp region identified as enriched for the respective epigenetic mark. “U” and “D” mean bins having biased interaction with upstream and downstream regions, respectively. “N” means bins without directionality bias. “X” means bins with any HMM state.


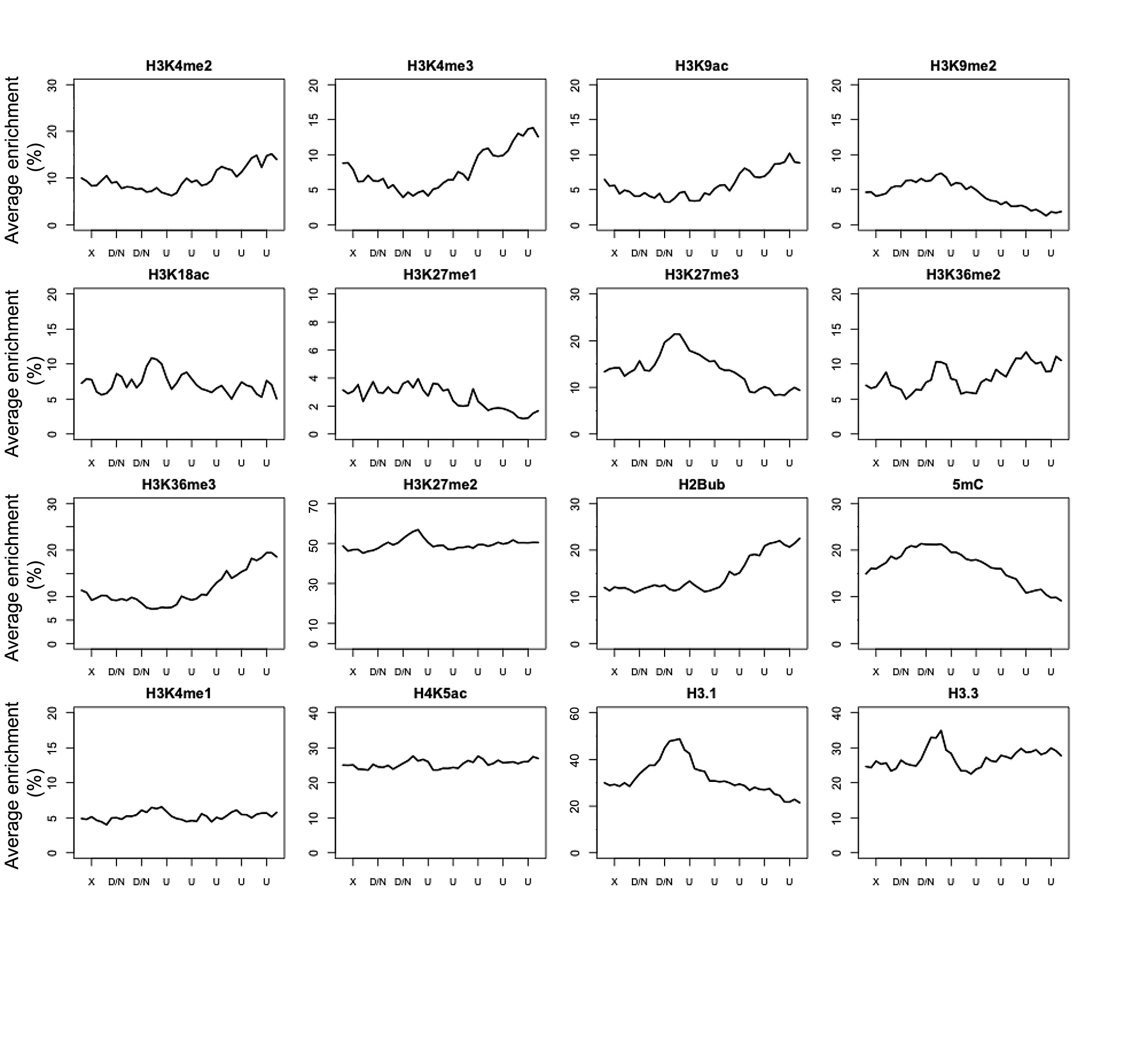


**Figure S26. Epigenetic marks associated with TAD-interior-like regions at the start of a “U” run.**

Average enrichment refers to fraction of each 400 bp region identified as enriched for the respective epigenetic mark. “U” and “D” mean bins having biased interaction with upstream and downstream regions, respectively. “N” means bins without directionality bias. “X” means bins with any HMM state.


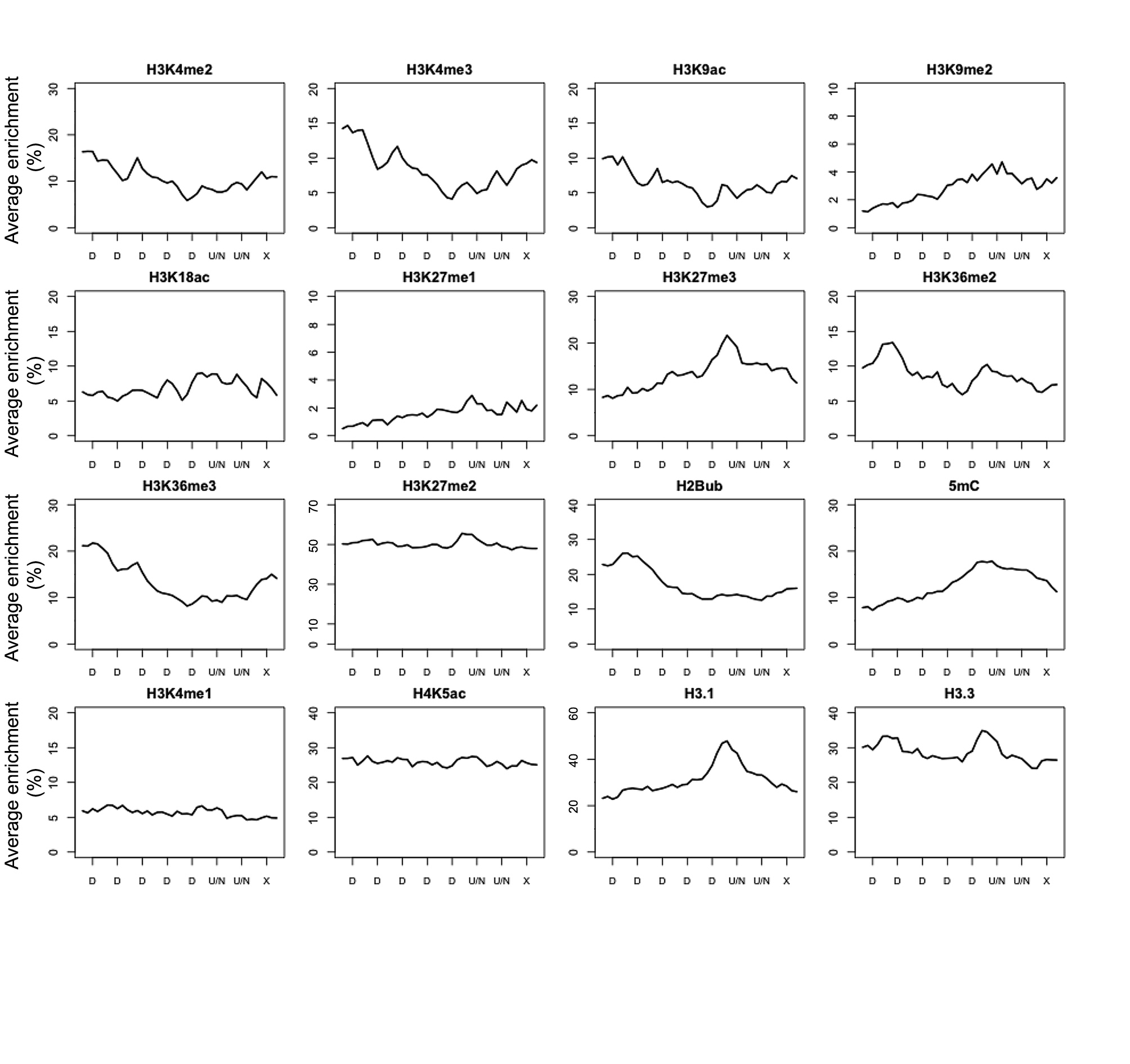


**Figure S27. Epigenetic marks associated with TAD-interior-like regions at the end of a of a “D” run.**

Average enrichment means the percentage of each 400 bp region claimed as enriched for the respective epigenetic mark. “U” and “D” mean bins having biased interaction with upstream and downstream regions, respectively. “N” means bins without directionality bias. “X” means bins with any type of HMM state.


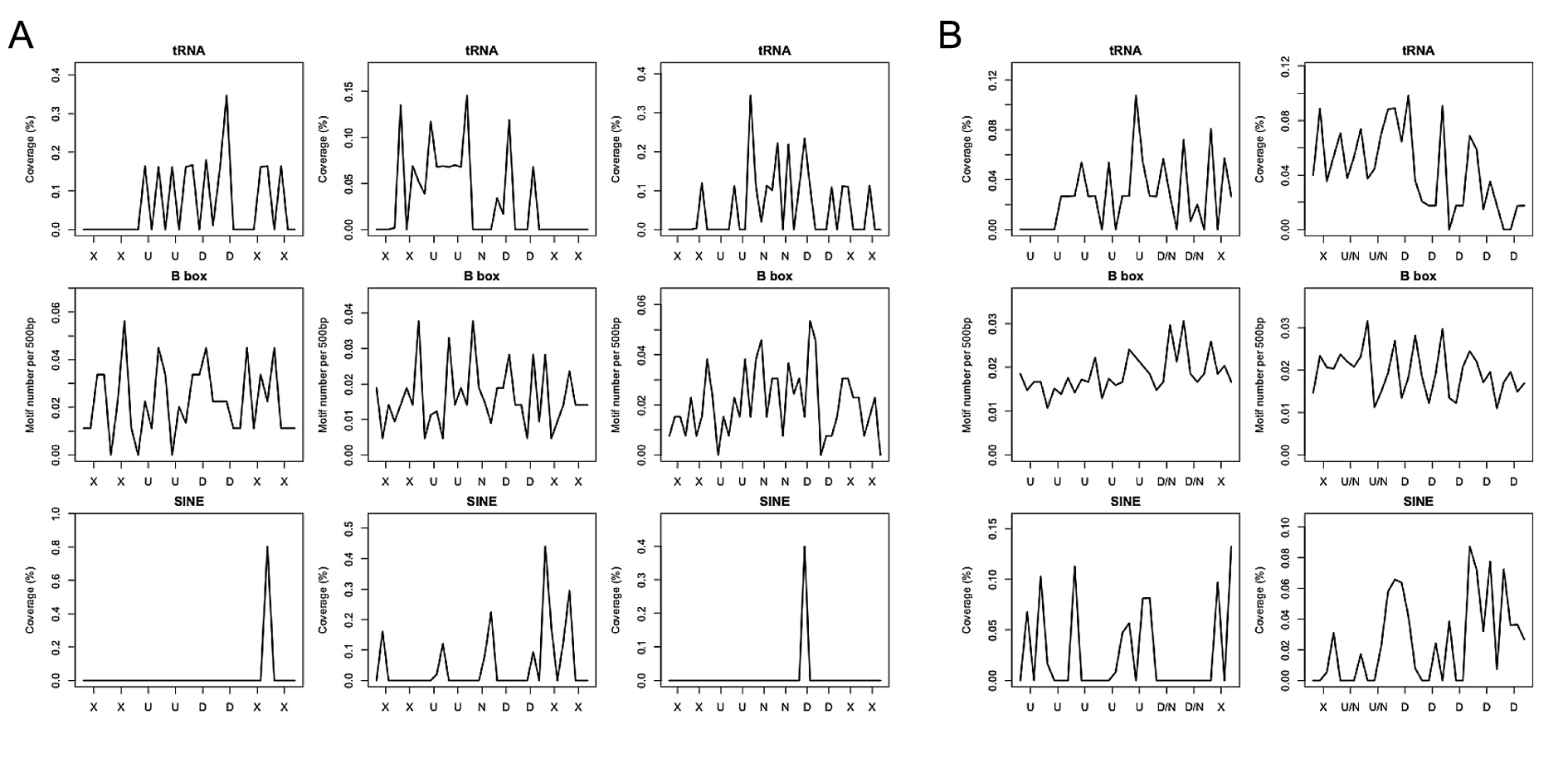


**Figure S28. Genomic features around insulator-like and TAD-boundary-like regions.**

The occurrence of tRNA, B box motif of tRNA promoter, and SINE transposons were plotted along insulator-like (A) and TAD-boundary-like (B) regions. Coverage means the average percentage of each 500 bp bin annotated with the respective sequence feature “U” and “D” mean bins having biased interaction with upstream and downstream regions, respectively. “N” means bins without directionality bias. “X” means bins with any type of HMM state.


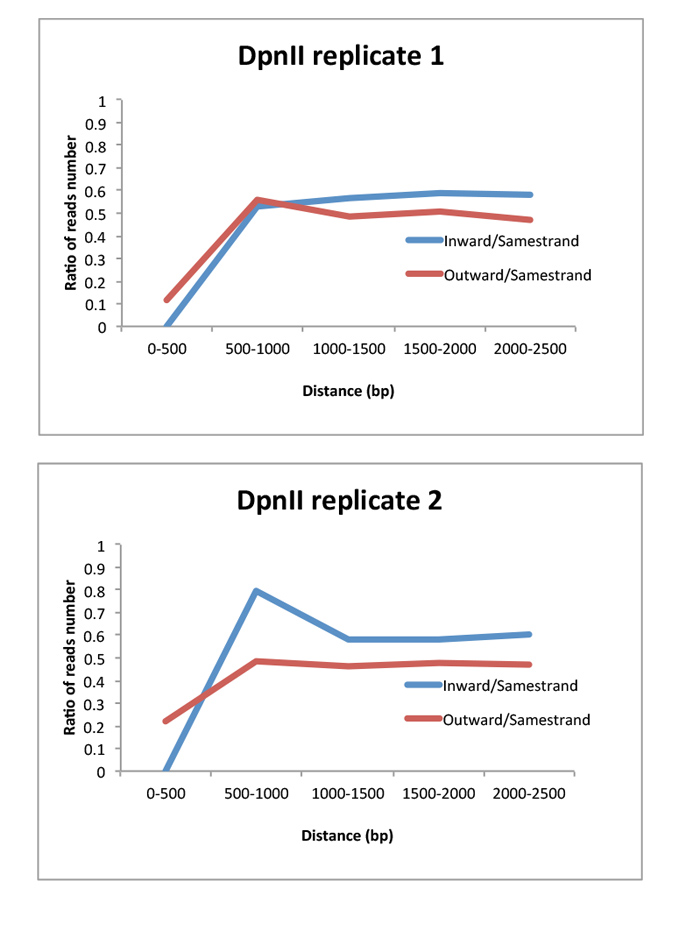


**Figure S29. Composition of types of read pairs as a function of the genomic distance between mapped loci.**

After filtering (see details in Methods), all retained read pairs with both ends mapped to the same chromosome were pooled and grouped according to the mapping mode (“Inward”, “Outward”, and “Samestrand”). The ratios of Inward/Samestrand and Outward/Samestrand reads were calculated based on a serious of distance ranges. For each distance range, only reads having the genomic distance within it were used for calculating the ratios. In an ideal situation, both Inward/Samestrand and Outward/Samestrand read ratios will be 0.5, as the ligation of digested DNA should have no bias on sequence direction with respect to the reference genome. However, self-ligation always produces “outward” type read pairs. Thus, if self-ligation products contribute substantially to a certain distance range, the Outward/
Samestrand ratio will be much higher than 0.5.


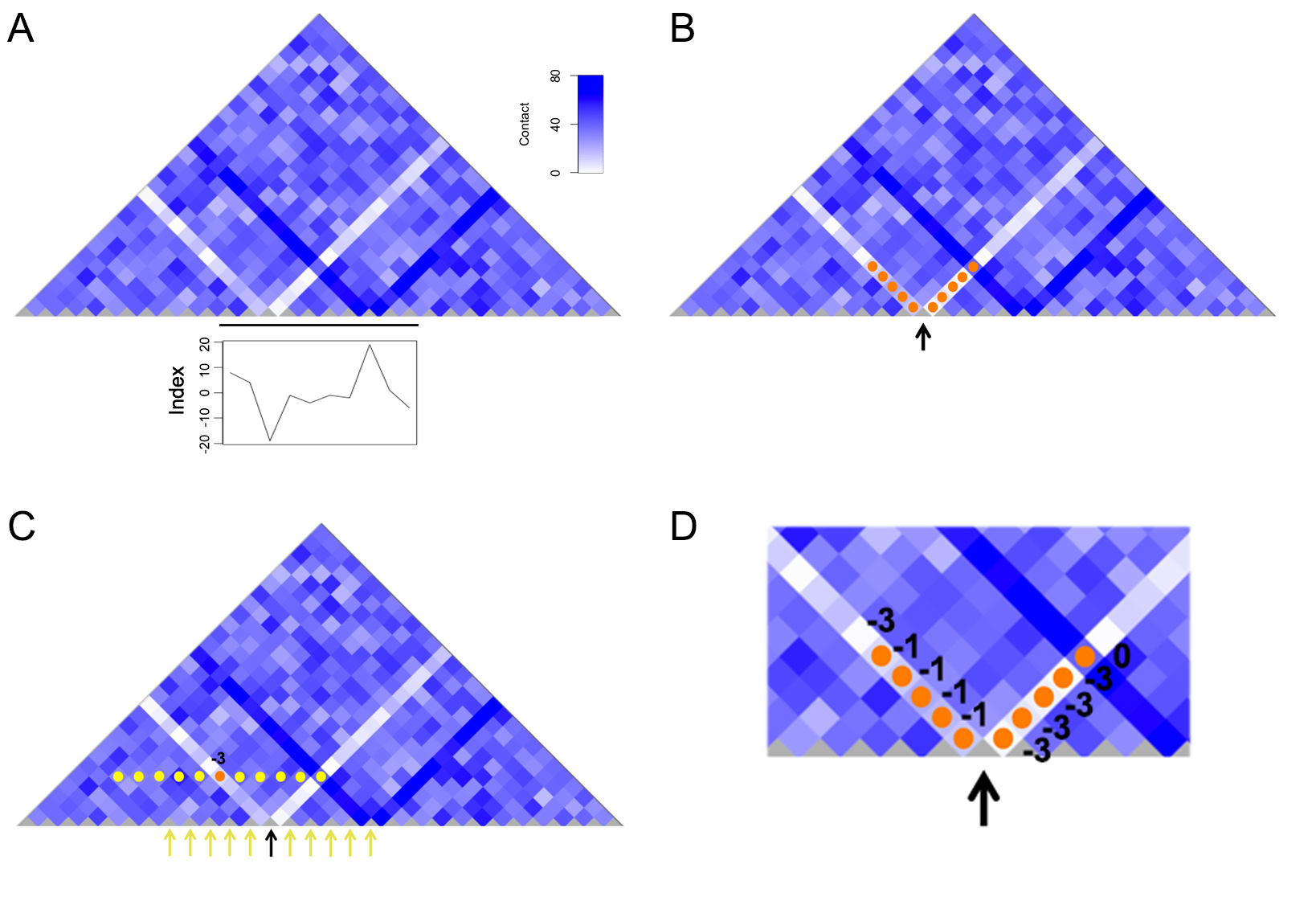


**Figure S30. Illustration of strip calling.**

(A) A simulated map where one bin has a negative strip and another bin a positive strip. The black line drawn below the map indicates bins from which index values were plotted. Different from those parameters used in analyzing the real Hi-C data, here for a bin of interest, only interaction with its 5 upstream and downstream bins are analyzed; while for the percentile check, only 10 neighboring bins are included. (B) For a bin of interest (arrow), its contact strengths with up to 5 interacting bins downstream or upstream were used to calculate the index value (orange dots). (C) For a particular contact strength (orange dot), it is compared with a pool of equivalent contact strength values (yellow dots) from 10 neighboring bins (yellow arrows), and a score was obtained. Here “-3” is given, since the contact strength belongs to the bottom 5% among all contact strength values considered. (D) This process was performed for all other contact strengths of the focal bin, and the sum of derived scores was defined as the index value of this bin.


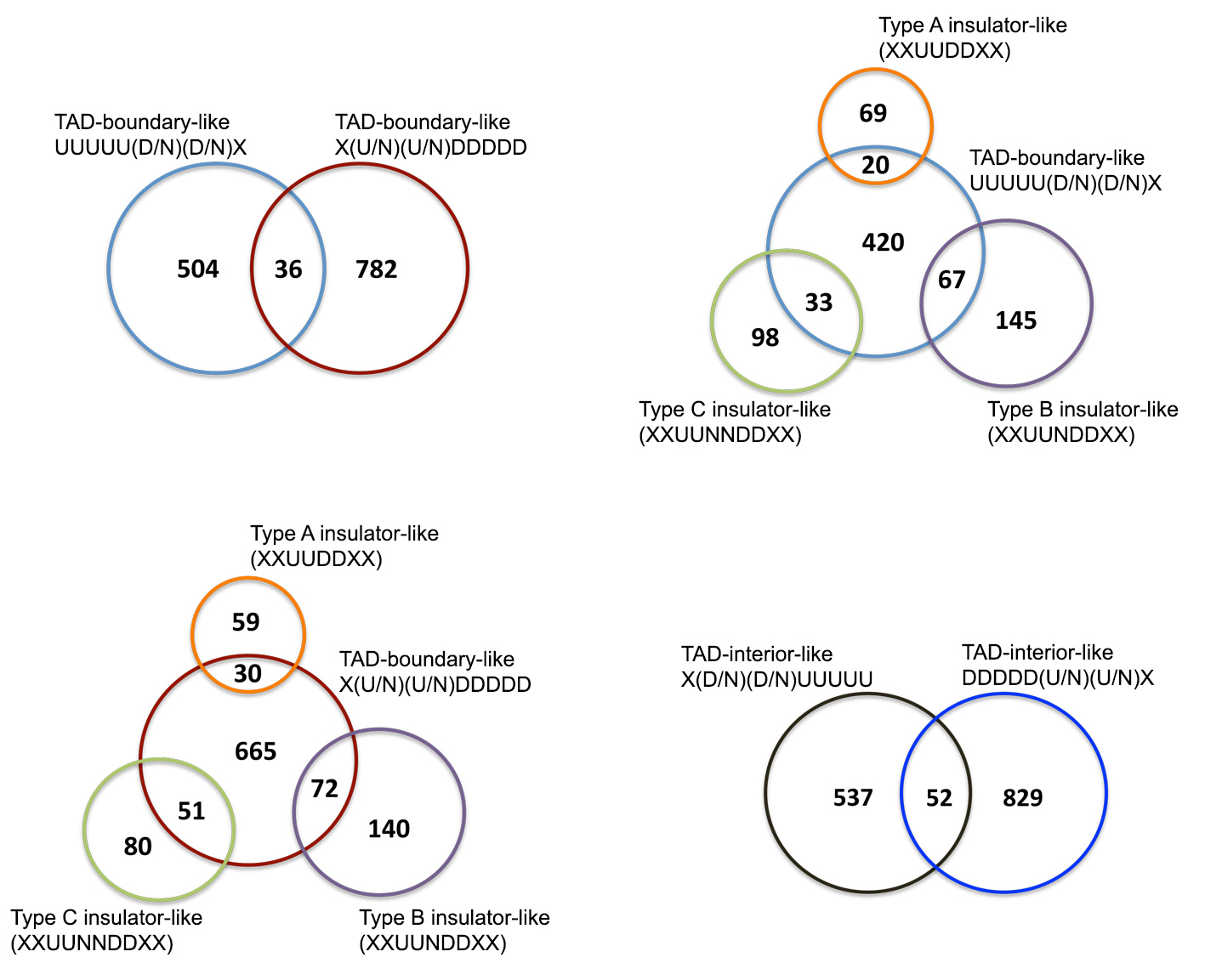


**Figure S31. Venn diagram of insulator-like, TAD-boundary-like, and TAD-interior-like regions.**
